# Supplementary material for: The oligosaccharyltransferase complex is an essential component of multiple myeloma plasma cells
Source: Mol Ther Oncol. 2025 Mar 8;33(2):200964. doi: 10.1016/j.omton.2025.200964 (PMC11978334; doi:10.1016/j.omton.2025.200964)
Supplement: Document S1. Figures S1–S11 and Table S1 [file mmc1.pdf]

OMTON, Volume *33*

Supplemental information

The oligosaccharyltransferase complex is an essential component of multiple myeloma plasma cells

Hong Phuong Nguyen, Enze Liu, Anh Quynh Le, Mahesh Lamsal, Jagannath Misra, Sankalp Srivastava, Harikrishnan Hemavathy, Reuben Kapur, Mohammad Abu Zaid, Rafat Abonour, Ji Zhang, Ronald C. Wek, Brian A. Walker, and Ngoc Tung Tran

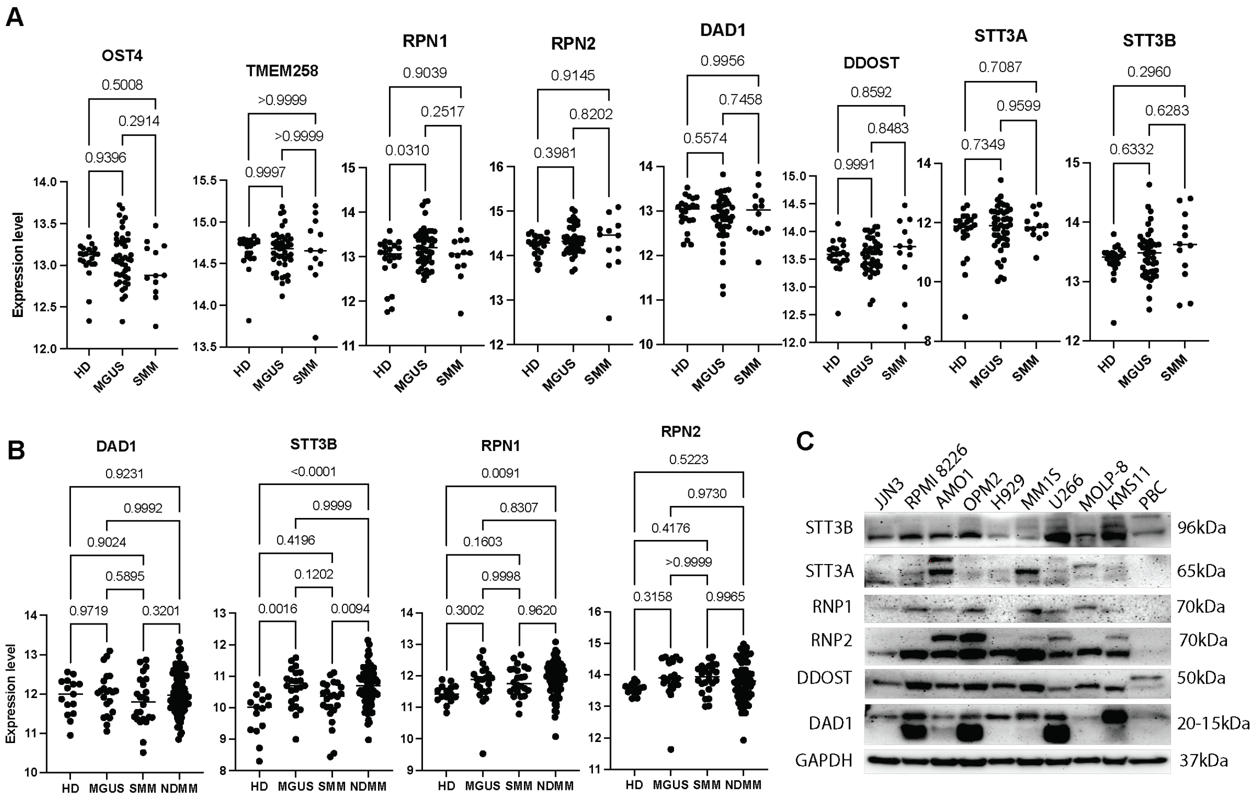

Figure S1. Expression level of OST's subunits in plasma cells isolated from healthy donor (HD), Monoclonal Gammopathy of Undetermined Significance (MGUS) patients, and Smoldering Multiple Myeloma patient (SMM). Data were extracted from microarray datasets: GSE5900 (A) and GSE6477 (B). (C) Western blot showing the protein levels of OST complex in different multiple myeloma cell lines and healthy peripheral blood cells.

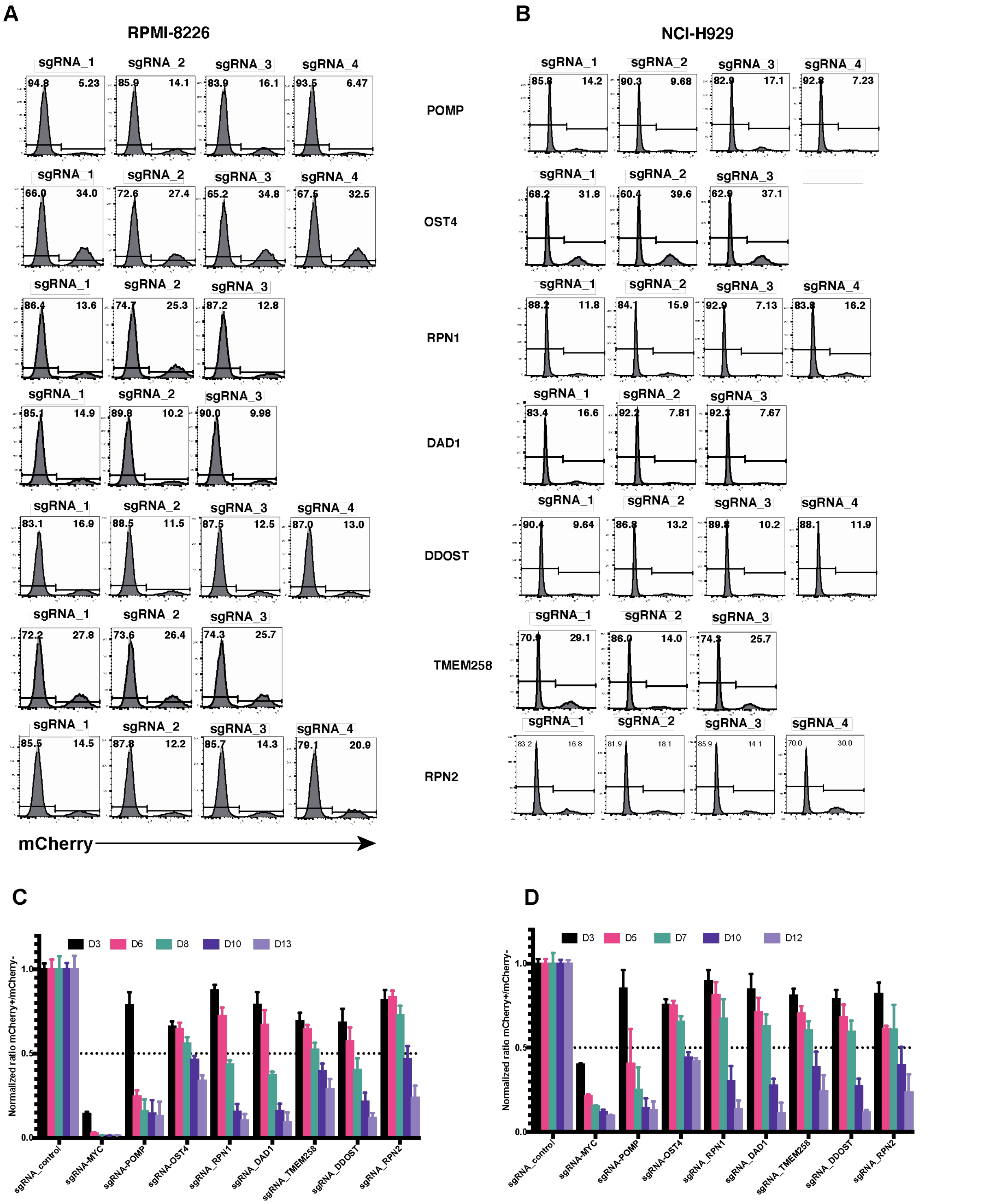

Figure S2. Histograms showing the proportion of mCherry+ cells (Cas9) in pre-gated BFP+ cells at day 12 after transducing with lentiviruses expressing indicated sgRNAs. Data from two cell lines were presented: RPMI-8226 (A) and NCI-H929 (B). Bar graphs summarize the normalized ratio of mCherry+/mCherry- in the BFP+ cells at different time points. Two cell lines were used in this experiment: RPMI-8226 (C) and NCI-H929 (D).

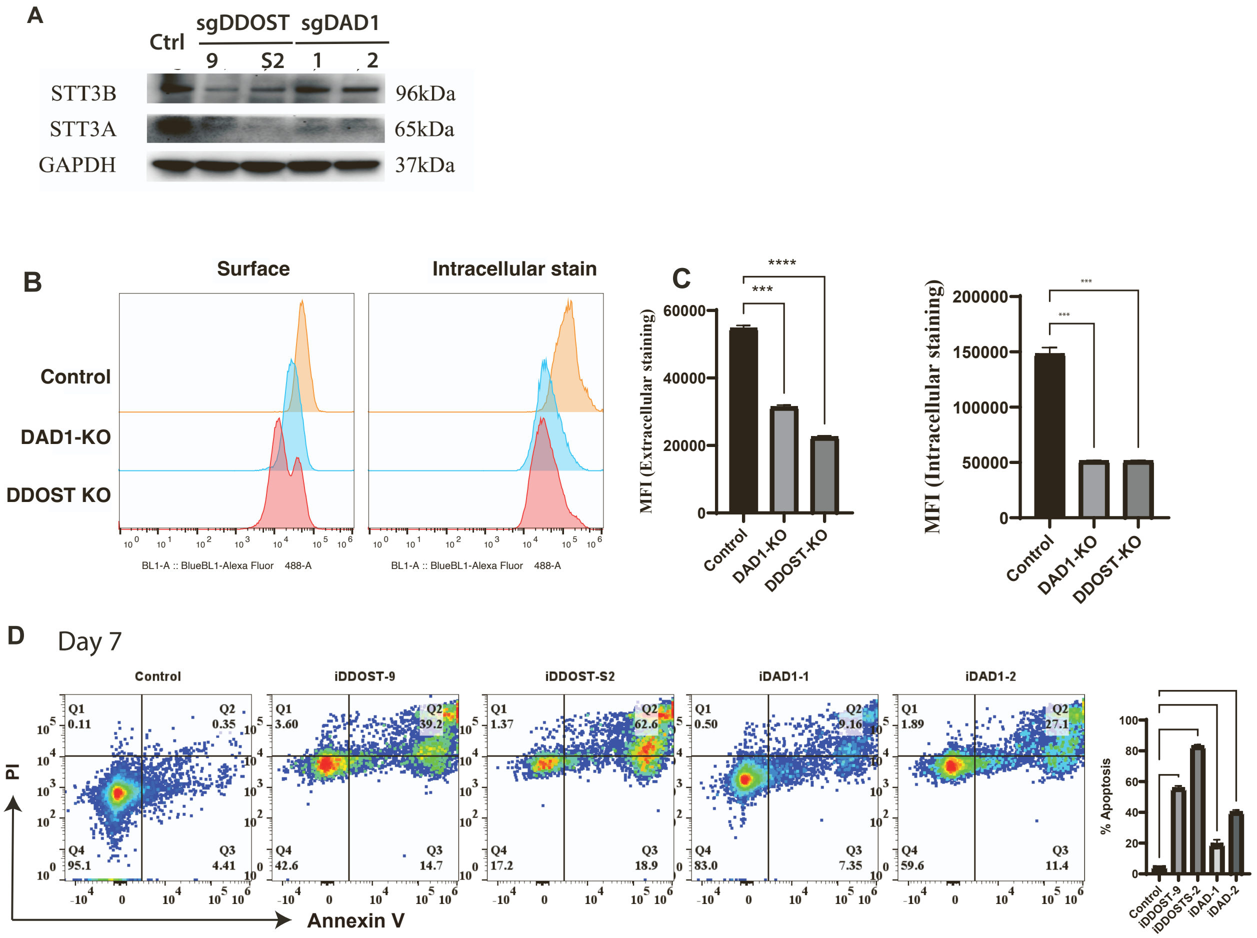

Figure S3. (A) Western blot showing the protein level of STT3A and STT3B in DDOST or DAD1-KO myeloma cells. We designed two sgRNAs per gene. (B) Histogram presenting the global surface and intracellular glycoproteins in DAD1 or DDOST-KO cells. (C) Data were presented as mean of fluorescent intensity (MFI). (D) Flow cytometry data showing the apoptosis of DAD1 or DDOST-KO cells using Annexin V staining (left). Data were summarized in the bar graph as means of percentage of apoptotic cells. *p<0.05, **p<0.01, ***p<0.001, and ****p<0.0001.

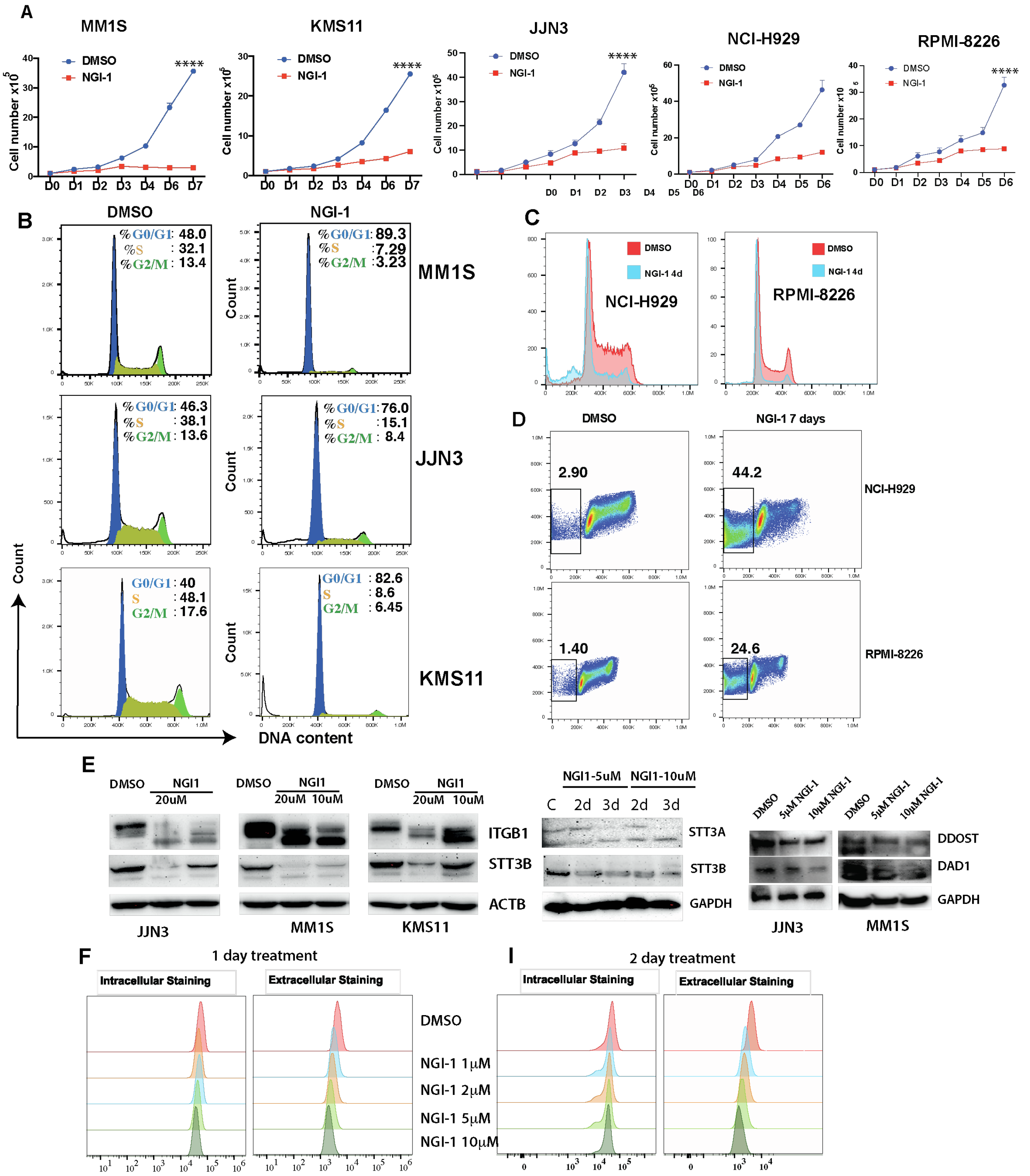

Figure S4. Dose-dependent curves presenting the sensitivity of TP53 mutant (A) and TP53 wildtype MM cell lines to NGI-1 treatment. NGI-1 GI50 (growth inhibition index) of these cell lines were calculated and presented in (C). Growth curves (D), cell cycle profiles (E), and cell death analysis (F) of NCI-H929 and RPMI-8226 upon being treated with DMSO or NGI-1 10mM. (G) western blot data from 3 different MM cell lines upon being treated with NGI-1. ITGB1 is known glycoprotein in MM cells, STT3A and B are catalytic subunit of the OST complex. GAPDH and Actin B (ACTB) are internal control. (H) Alexa-488-conjugated lectin staining for MM1S cells treated with different concentrations of NGI-1 for 2 days. (I) Flow cytometry data

showing the lectin staining for control and DAD1 or DDOST KO cells. cells were analyzed by flow cytometry to detect global surface (left) and intracellular (right) N-glycoproteins. (Growth curve (J), apoptosis (K), and cell cycle profiles (L) of peripheral blood CD34+ cells upon treated with NGI-1

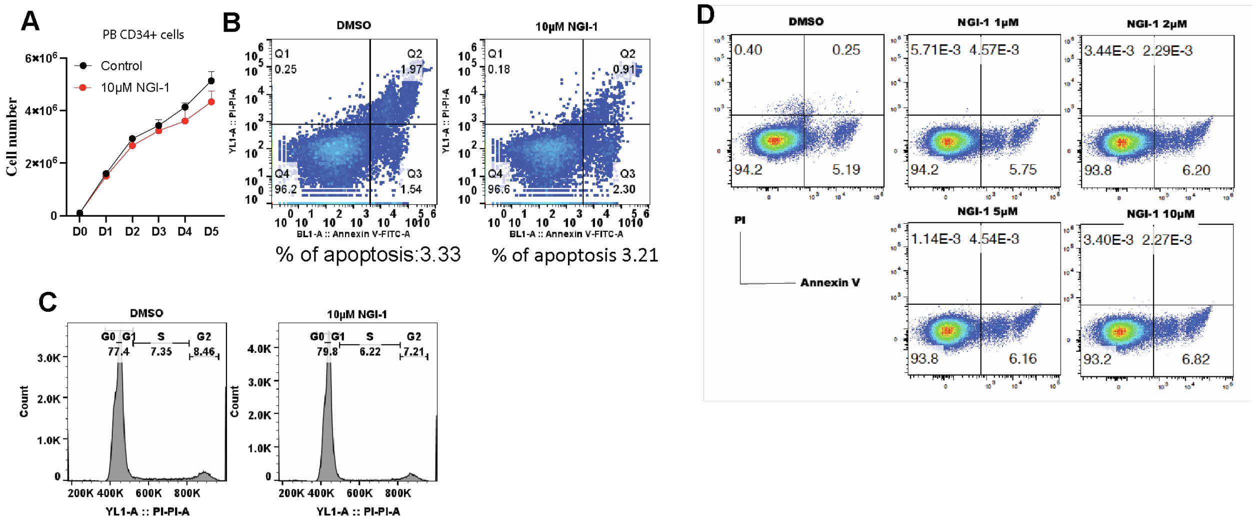

Figure S5. Growth curves (A), apoptosis (B), and cell cycle profile of human CD34+ cells upon treating with NGI-1 or DMSO control. (D) Apoptosis profile of human primary fibroblast cells upon treated with different doses of NGI-1 for 3 days.

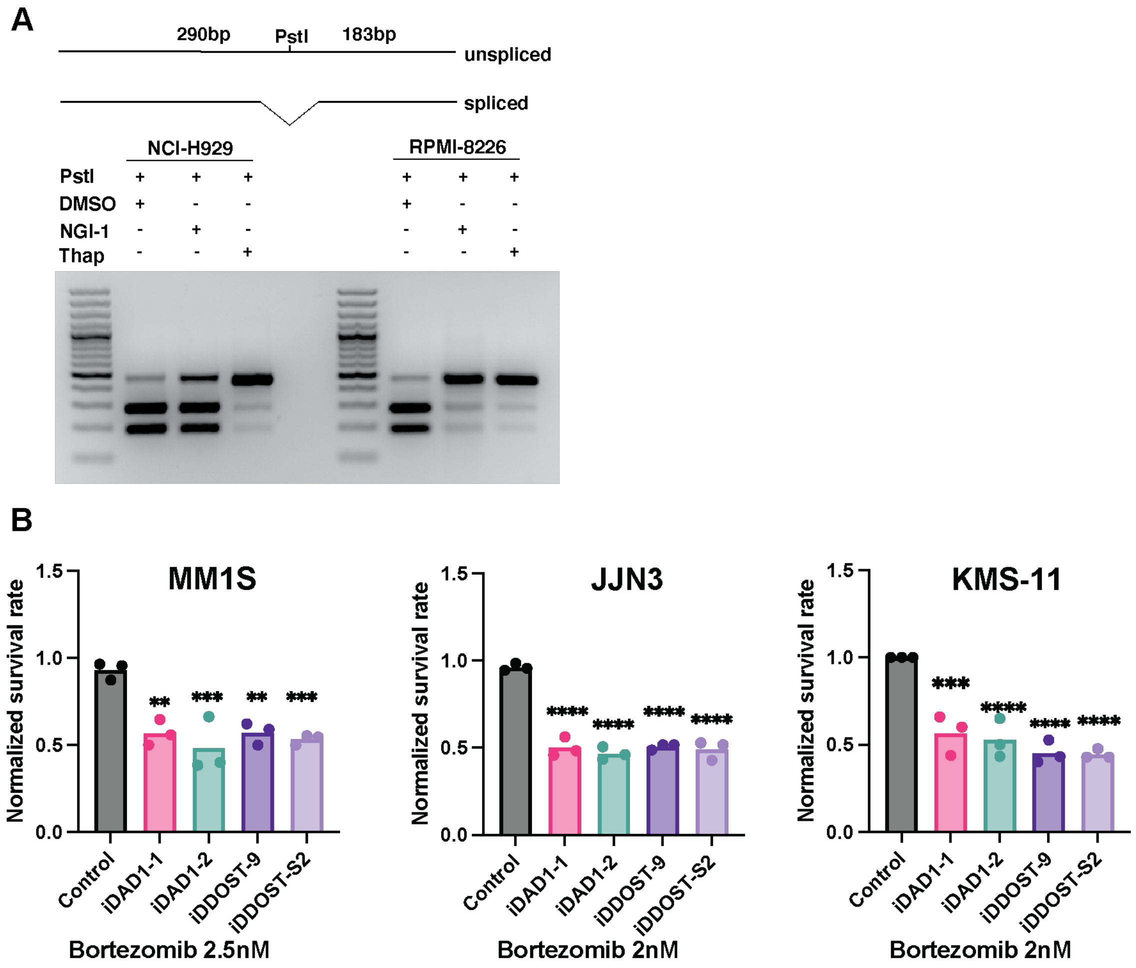

Figure S6. (A) XBP1 splicing assay indicating the ER stress status of MM cell lines treated with NGI-1. PstI is restriction enzyme site located in the XBP1 unspliced transcript. Upon ER stress induction, XBP1 will be spliced and removed the PstI site. Thapsigagin servers as positive control for ER stress inducer. Agarose gel showing the PCR products after digesting with PstI. (B) Bar graphs presenting the normalized survival of MM cell lines upon knocking out of DAD1 or DDOST in the presence of indicated bortezomib doses.

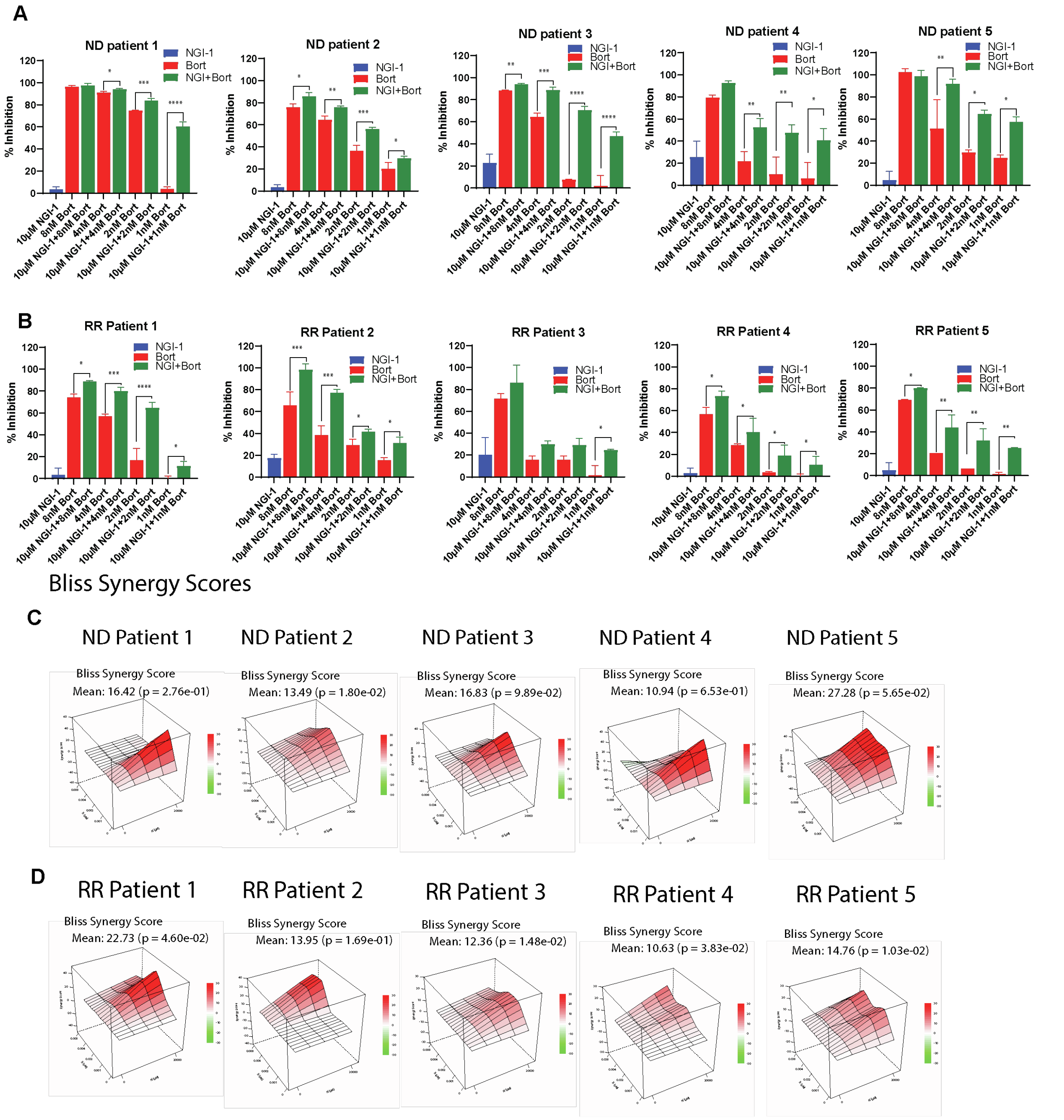

Figure S7. Bar graphs summarize the cell viability assay of newly diagnosed (ND) patients (A) and relapsed/refractory (RR) patients (B) upon being treated with NGI-1 and different indicated doses of bortezomib. Data from each patient is the mean of technical tripreplicates. *p<0.05, **p<0.01, ***p<0.001, ****p<0.0001. Graphs presenting the means of Bliss Synergy scores in each ND (C) or RR (D) patients upon being treated with NGI-1 and bortezomib.

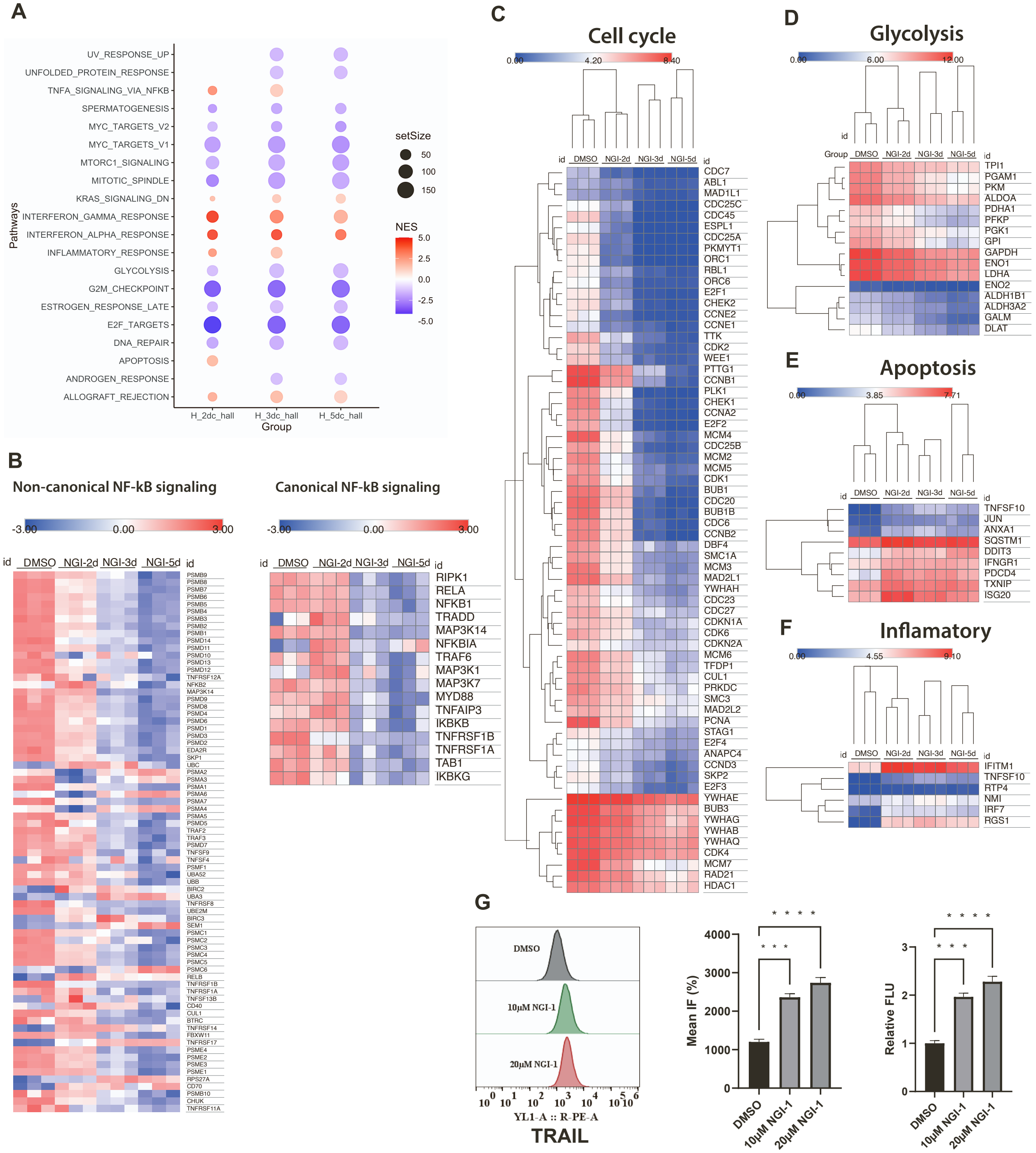

Figure S8. (A) pathway analysis of differential expressed genes in MM1S cell upon being treated with NGI-1 for different days. Heatmap presenting the differential expressed genes in NF-kB signaling pathways (B), Cell cycle (C), Glycolysis (D), Apoptosis (E), and Inflammatory (F) upon being treated with NGI-1. (G) Flow cytometry (left) data depicts the expression of TRAIL in MM cells treated with NGI-1 at different doses for 3 days. Bar graphs (right) summarize the mean fluorescent intensity and relative fluorescent units calculated from C.

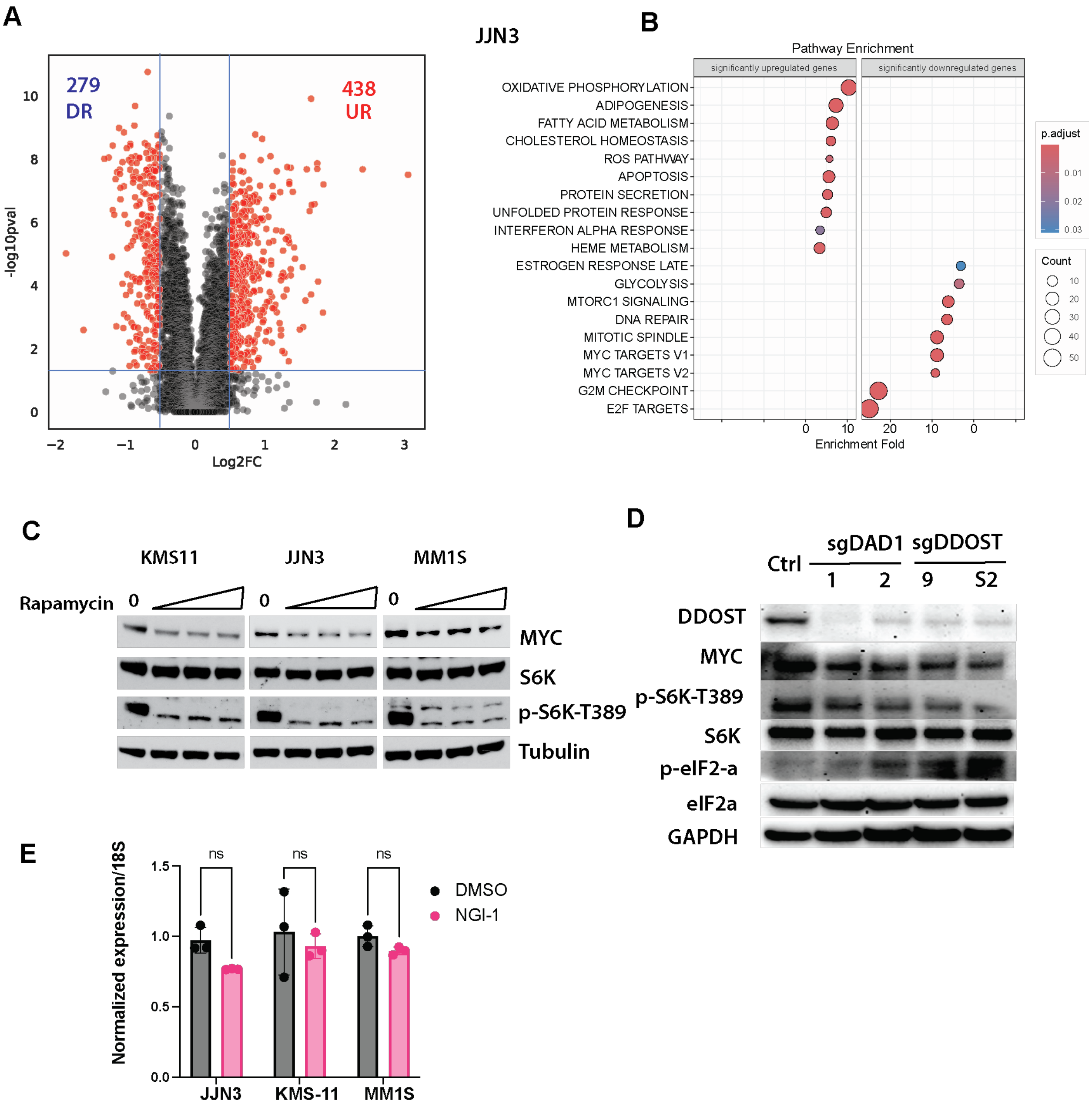

Figure S9. (A) Volcano plot showing differentially expressed proteins (DEP) in JJN3 cells upon treated with NGI-1, DR (downregulated), UR (upregulated). (B) Pathway analysis of DEPs. (C) Western blot showing the activity of mTOCR1 pathway and MYC upon treated with rapamycin (50nM, 100nM and 200nM) at different concentrations. Tubulin was used as a loading control. (D) Western blot showing the protein level of MYC and activity of the mTOCR1 pathway in DAD1 or DDOST-KO myeloma cells. (E) mRNA level of MYC in MM cells upon treating with NGI-1 10μM.

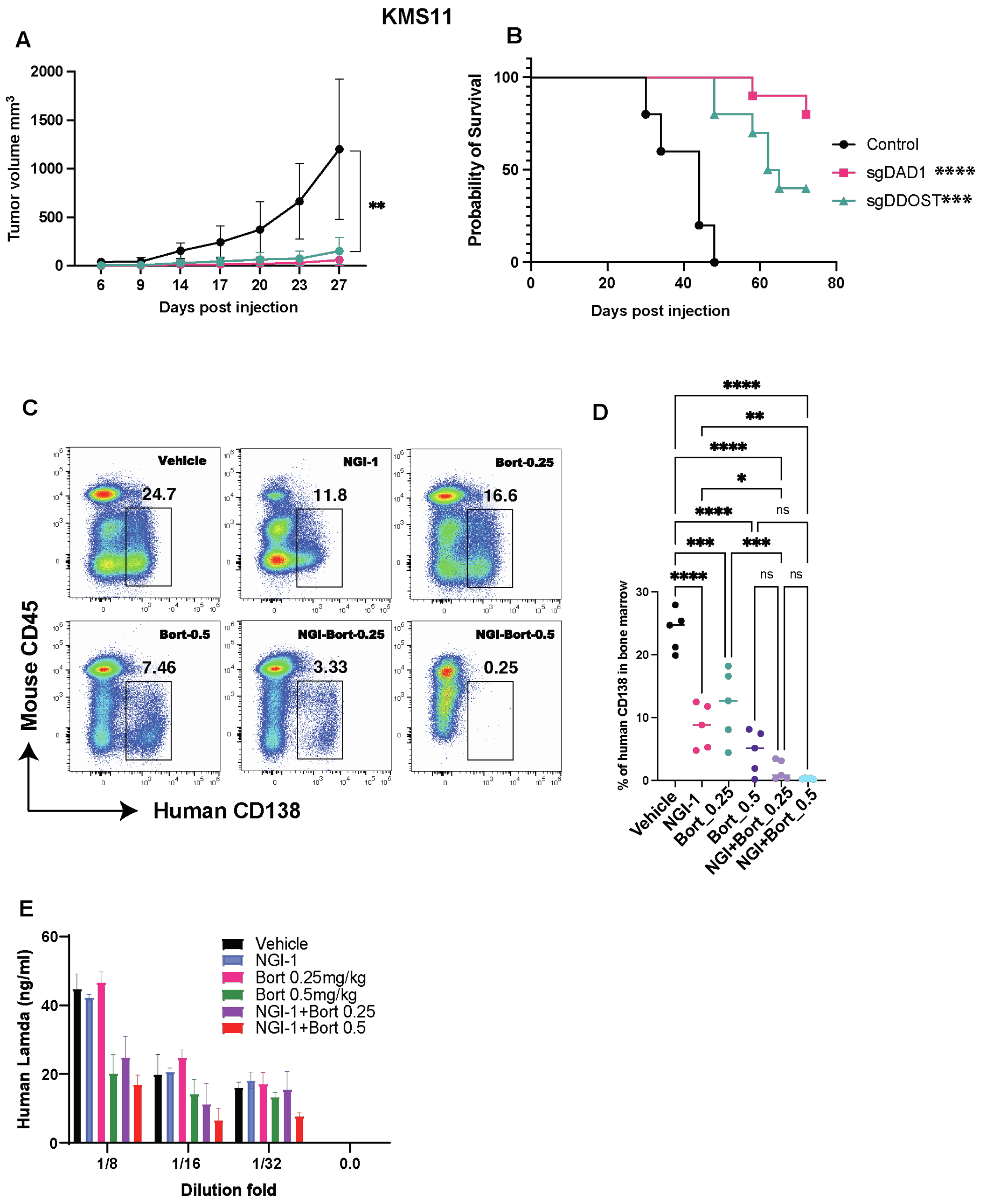

Figure S10. (A) Tumor volumes and Kaplan survival curves of mice subcutaneously implanted with KMS11 control or DAD1/DDOST KO cells. (C) Flow cytometry presenting the percentages of human CD138+ cells in the bone marrow of mice intravenously injected with MM1S cells after different treatment conditions. (D) Graph summarizes data in C from different drug treatment groups (N=5). (E) ELISA showing the level of human lambda levels in peripheral blood of mice treated with different conditions (different dilution of serum) at termination of experiment (4 weeks post-implantation).

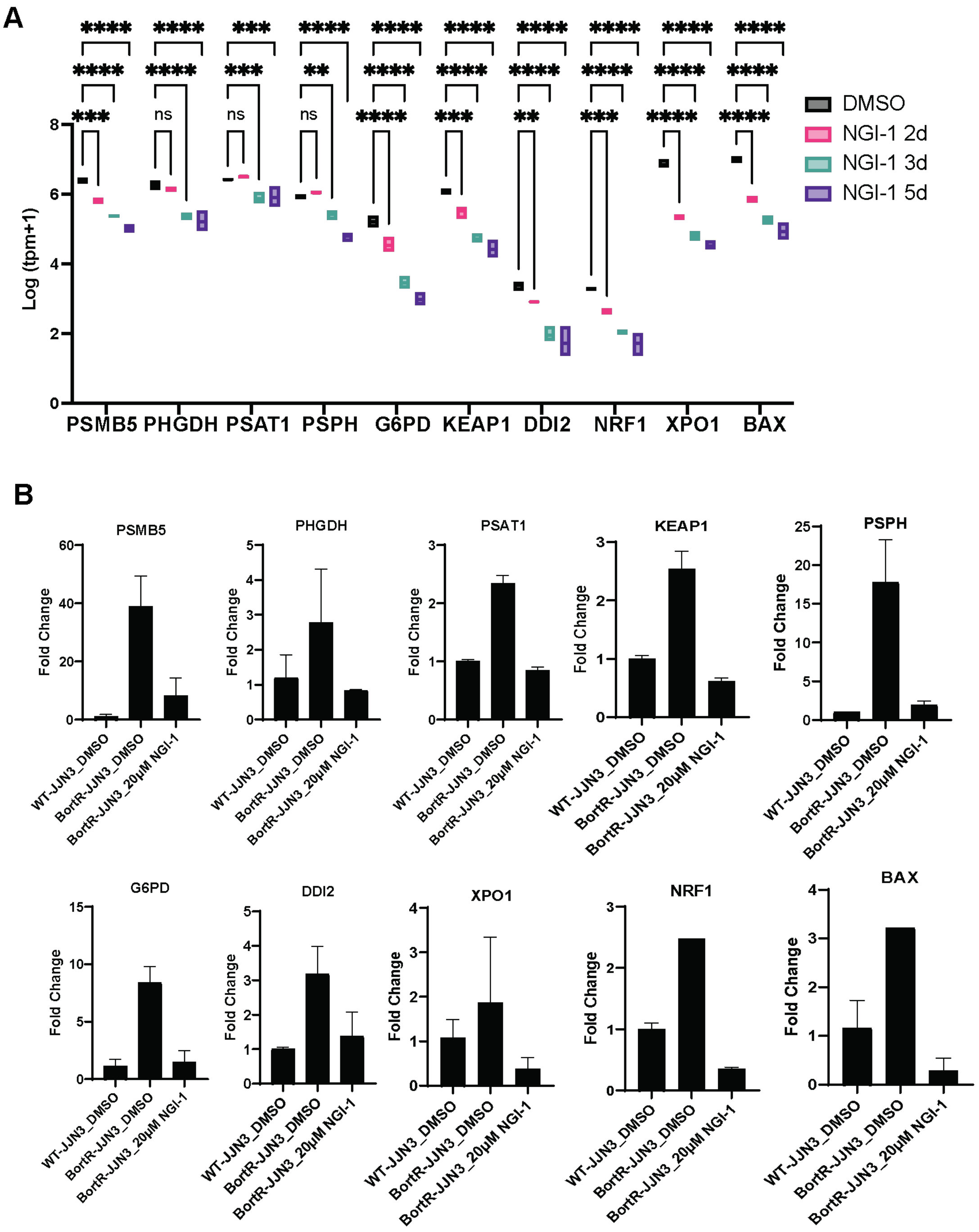

Figure S11. Expression levels of genes that are known to associated with bortezomib resistant phenotype were extracted from RNA-seq data (A) of MM1S cells treated with NGI-1 as indicated. (B) Real-time PCR data showing the expression of indicated genes in parental JJN3 and bortezomib resistant JJN3 (BortR-JJN3) cells upon treating with NGI-1.

Table S1. Antibodies used in this study		
DDOST Polyclonal antibody	14916-1-AP	Protein tech
STT3A Polyclonal antibody	12034-1-AP	Protein tech
STT3B Polyclonal antibody	15323-1-AP	Protein tech
DAD1 Polyclonal antibody	10531-1-AP	Protein tech
Beta Actin Monoclonal antibody	66009-1-Ig	Protein tech
GAPDH Monoclonal antibody	60004-1-Ig	Protein tech
HRP-conjugated Affinipure Goat Anti-Mouse IgG(H+L)	SA00001-1	Protein tech
HRP-conjugated Affinipure Goat Anti-Rabbit IgG(H+L)	SA00001-2	Protein tech
ITGB1 ANTIBODY	PA529606	Thermo
Goat Anti-Mouse IgG(H+L), Human ads-HRP	1031-05	SouthernBiotech
Goat Anti-Rabbit IgG-HRP	4030-05	SouthernBiotech
c-MYC(D84C12) Rabbit mAb	#5605	Cell signaling
S6K (49D7)	#2708	Cell Signaling
p-S6K-T389 (108D2)	#9234	Cell Signaling
p-eIF2a-S51 (D9G8)	#3398	Cell Signaling
eIF2a	#9722	Cell Signaling
CD138-APC (MI15)	#356506	Biolegend
PE/Cy7 anti-mouse CD45 (Clone 30-F11)	#103113	Biolegend
PE anti-human CD253 (TRAIL) Antibody	308206	Biolegend
