# Supplementary material for: The oligosaccharyltransferase complex is an essential component of multiple myeloma plasma cells
Source: Mol Ther Oncol. 2025 Mar 8;33(2):200964. doi: 10.1016/j.omton.2025.200964 (PMC11978334; doi:10.1016/j.omton.2025.200964)
Supplement: Document S2. Article plus supplemental information [file mmc2.pdf]

Molecular Therapy
Oncology
Original Article

The oligosaccharyltransferase complex is an essential component of multiple myeloma plasma cells

Hong Phuong Nguyen,[1] Enze Liu,[2] Anh Quynh Le,[1] Mahesh Lamsal,[1] Jagannath Misra,[4] Sankalp Srivastava,[1] Harikrishnan Hemavathy,[1] Reuben Kapur,[1] Mohammad Abu Zaid,[2] Rafat Abonour,[2] Ji Zhang,[1,4] Ronald C. Wek,[4] Brian A. Walker,[2,3] and Ngoc Tung Tran[1]

[1]Herman B. Wells Center for Pediatric Research, Department of Pediatrics, Indiana University School of Medicine, Indianapolis, IN, USA; [2]Melvin and Bren Simon Comprehensive Cancer Center, Division of Hematology and Oncology, School of Medicine, Indiana University, Indianapolis, IN, USA; [3]Center for Computational Biology and Bioinformatics, School of Medicine, Indiana University, Indianapolis, IN, USA; [4]Department of Biochemistry and Molecular Biology, Indiana University School of Medicine, Indianapolis, IN 46202, USA

Multiple myeloma (MM) is an incurable malignancy characterized by mutated plasma cell clonal expansion in the bone marrow, leading to severe clinical symptoms. Thus, identifying new therapeutic targets for MM is crucial. We identified the oligosaccharyltransferase (OST) complex as a novel vulnerability in MM cells. Elevated expression of this complex is associated with relapsed, high-risk MM, and poor prognosis. Disrupting the OST complex suppressed MM cell growth, induced cell-cycle arrest, and apoptosis. Combined inhibition with bortezomib synergistically eliminated MM cells *in vitro* and *in vivo*, via suppressing genes related to bortezomib-resistant phenotypes. Mechanistically, OST complex disruption downregulated MM pathological pathways (mTORC1 pathway, glycolysis, MYC targets, and cell cycle) and induced TRAIL-mediated apoptosis. Notably, MYC translation was robustly suppressed upon inhibiting the OST complex. Collectively, the OST complex presents a novel target for MM treatment, and combining its inhibition with bortezomib offers a promising approach for relapsed MM patients.

INTRODUCTION

Multiple myeloma (MM) is the second-most prevalent hematological malignancy, characterized by the clonal expansion of malignant plasma cells within the bone marrow resulting in the overproduction of abnormal immunoglobulins. The resultant overcrowding transforms the bone marrow microenvironment, leading to severe clinical manifestations, including bone lesions, hypercalcemia, anemia, immune suppression, and multiple organ failure.[1,2] This heterogeneous disease exhibits genetic defects linked to chromosomal abnormalities and mutations in tumor-suppressor and oncogenes.[3] Despite advancements in therapies, relapsed/refractory (RR) MM remains incurable, underscoring the urgent need for novel therapeutic targets.

N-linked glycosylation, a prominent post-translational modification, profoundly influences the function of secretory and membrane-bound proteins.[4] Its critical roles encompass cell signaling, protein folding, degradation, trafficking, and cell-cell interactions.[5,6] The catalysis of glycan transfer to nascent proteins is orchestrated by the multi-subunit oligosaccharyltransferase (OST) complex.[7–10] Mammalian cells harbor two distinct OST complexes, OST-A (with catalytic subunit STT3A) and OST-B (with catalytic subunit STT3B), both sharing six accessory subunits: RPN1, RPN2, OST4, DAD1, TMEM258, and DDOST.[11–13] Aberrant OST complex expression has been implicated in solid tumors,[14,15] yet its involvement in MM pathogenesis remains elusive.

Here, we demonstrated that the OST complex is a novel vulnerability in MM cells. Analysis of publicly available genomic data revealed abundant expression of all shared OST subunits in MM cell lines and patient-derived cells. Elevated expression of the OST complex strongly correlated with RR MM and poor survival outcomes. This correlation prompted the hypothesis that the OST complex plays a pivotal role in MM pathology. We demonstrated that disruption of the OST complex, through genetic or pharmacological approaches, impedes MM cell growth, induces cell death, and arrests the cell cycle. Notably, OST complex inhibition sensitizes MM cell lines and MM patient's cells, including bortezomib-resistant cells, to bortezomib treatment by suppressing genes known to be associated with the bortezomib-resistant phenotype. We found a profound suppression of MM tumor growth *in vivo* in a xenograft model using this combination treatment. Mechanistically, OST complex disruption resulted in a significant downregulation of pathologically relevant pathological signatures of MM cells (both transcript and protein level), concomitant with the induction of apoptosis and inflammatory pathways. We

Received 24 September 2024; accepted 5 March 2025;
https://doi.org/10.1016/j.omton.2025.200964.

Correspondence: Ngoc Tung Tran, Herman B. Wells Center for Pediatric Research, Department of Pediatrics, Indiana University School of Medicine, Indianapolis, IN, USA.
E-mail: tungtran@iu.edu

Published by Elsevier Inc. on behalf of The American Society of Gene and Cell Therapy.
This is an open access article under the CC BY license (http://creativecommons.org/licenses/by/4.0/).

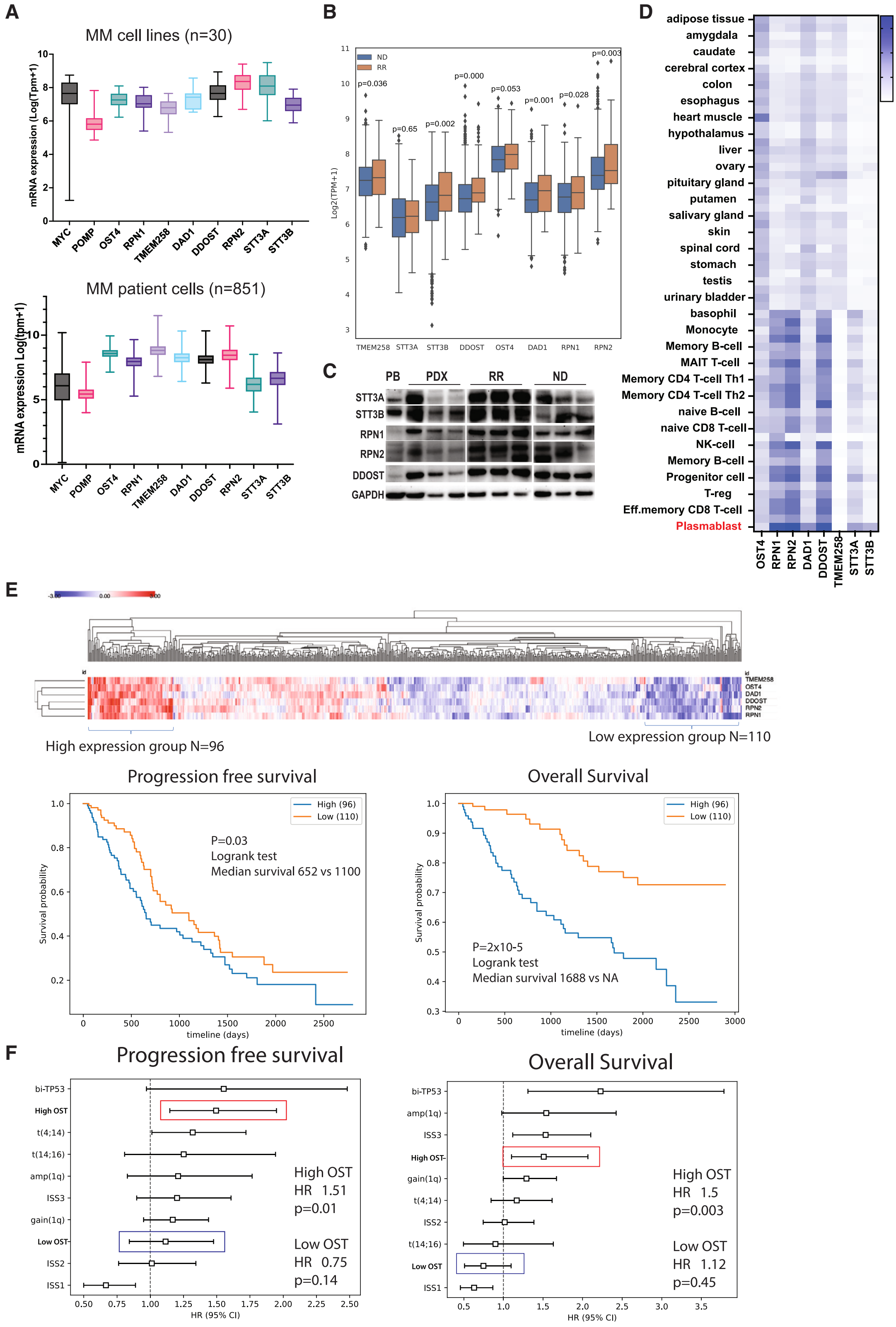

(legend on next page)

also found that the translation of MYC was significantly reduced upon inhibition of the OST complex.

In summary, our study provides pioneering preclinical evidence establishing the OST complex as a crucial player in MM pathology, representing both a genetic and therapeutic vulnerability, particularly in bortezomib-resistant MM cells.

RESULTS

The OST complex is highly expressed in MM cells but not in normal tissues

Previously, we found that the OST complex is essential to promote the survival, proliferation, and differentiation of murine B cells into plasma cells.[16,17] To investigate the functions of the OST complex in the context of MM, we examined the expression levels of OST complex components in MM cell lines using data from the Cancer Cell Line Encyclopedia (CCLE). We observed that MM cell lines exhibit notably elevated expression of shared (*RPN1, RPN2, OST4, DAD1, TMEM258,* and *DDOST*) and catalytic (*STT3A* and *STT3B*) subunits of the OST complex. The expression levels of these subunits were comparable with those of *MYC*[18] and *POMP*,[19,20] two known oncogenes in MM (Figure 1A, top panel). Subsequently, we conducted a comprehensive analysis of transcriptomic database of MM patients from the Multiple Myeloma Research Foundation CoMMpass study and found these subunits were also highly expressed in MM patient cells (Figure 1A, bottom). Interestingly, relapsed MM patients (RR) expressed significantly higher expression levels of the OST complex than newly diagnosed patients (ND) (Figure 1B). To evaluate the expression of the OST subunits across MM stages, we analyzed microarray data from published datasets: GSE5900[21–23] and GSE6477.[24–27] We found that OST subunits were highly expressed in plasma cells isolated from healthy donors and MM patients at various stages. *DDOST, STT3B, RPN2*, and *RPN1* exhibited an increased expression trend in MGUS upon disease progression, but not significant (Figures S1A and S1B). We also found that the protein levels of the OST subunits in MM cell lines (Figure S1C), MM patients, and patient-derived xenograft samples (Figure 1C) were higher than those in peripheral blood from healthy donor.

Extracting the expression of the OST complex in normal tissues from the Human Protein Atlas database revealed that the OST complex was generally expressed higher in blood cells than other normal tissues. Among blood cell types, plasmablasts exhibited the highest expression of the OST complex (Figure 1D). These data indicate that the OST complex might be a common vulnerability in both myeloma and plasma cells.

When grouping patients based on the expression level of the OST complex, we observed that patients exhibiting high expression of OST complex components were significantly associated with worse progression-free and overall survival (Figure 1E). We also performed multivariate cox proportional hazard regression to compare the hazard ratio (HR) of patients with high and low expression level of the OST complex to the common high-risk genomic factors. We found that high expression level of the OST complex was significantly associated with high risk in overall survival (HR = 1.51, $p = 0.01$) and progression-free survival (HR = 1.5, $p = 0.003$) along with well-known high-risk factors in MM such as biallelic TP53 inactivation (bi-TP53), t(4; 14), t(4; 16), amplification or gain of 1q, and international staging system 3 (ISS3, high risk) (Figure 1F). By comparison, low expression level of the OST complex did not demonstrate any statistical significance associated with high risk. Collectively, these findings suggest that the OST complex might be essential for myeloma pathology.

Deletions of the OST-shared subunits impair the cell survival and growth of MM cells

Mammalian cells have two OST complexes, designated OST-A and OST-B; therefore, to simultaneously target both complexes, we deleted their shared subunits *OST4, RPN1, RPN2, TMEM258, DAD1,* and *DDOST*. To this end, we devised a dual reporter system to assess the functionality of specific genes within MM cell lines (Figure 2A). Briefly, we employed the CrispRGold program[16] to design three to five single-guide RNAs (sgRNAs) targeting each of these shared subunits. Subsequently, these sgRNAs were cloned into a lentiviral vector expressing blue fluorescent protein (BFP), serving as the first reporter. Concurrently, we established MM cell lines (NCI-H929 and RPMI-8226) with stable expression of Cas9-T2A-mCherry (Cas9-MM), which acted as the second reporter. We combined parental MM cells with Cas9-MM cells in a 1:1 ratio and cultured them in a single well of a 96-well plate. The resulting cell mixture was transduced with lentivirus carrying the respective sgRNAs. The infected cells (BFP^+) were analyzed using flow cytometry at different time points to calculate the ratio of $mCherry^+BFP^+$ cells (knockout cells) to $mCherry^-BFP^+$ cells (non-edited cells). This ratio served as an indicator for the knockout impact of a gene on MM cell lines. As positive controls, we included sgRNAs targeting *MYC* and *POMP*. As anticipated, in two distinct cell lines, the knockout of

Figure 1. The OST complex is associated with poor prognosis in MM

(A) Bar graphs show expression of indicated genes in MM cell lines ($n = 30$, top) from Cancer Cell Line Encyclopedia and in MM patients' cells ($n = 851$, bottom) from the Multiple Myeloma Research Foundation CoMMpass study. Boxes represent mean of expression level. (B) Bar graph exhibits expression of the shared subunits of the OST complex in newly diagnosed (ND) patient and relapsed/refractory (RR) patients. p values are indicated. (C) Western blot showing the expression of the OST subunits in peripheral blood cells (PB), MM cells isolated from ND/RR patients and rabbit bones engrafted with patient MM cells (PDX). (D) Expression level of the OST subunits in normal tissues (cell types are indicated) extracted from the Human Protein Atlas database. (E) MM patients were grouped based on the expression level of the OST complex: high expression group ($N = 96$) and low expression group ($N = 110$). The progression-free and overall survival of these patient groups are presented. p values and test are indicated. (F) Multivariate analysis showing hazard ratio (HR) of common high-risk genomic factors: biallelic deletion of TP53 (bi-TP53), translocation t(4; 14) and t(14; 16), amplification of 1q, gain of 1q, international staging system 1, 2 (ISS1 and ISS2, low risk), and 3 (ISS3, high risk). CI stands for confidence interval.

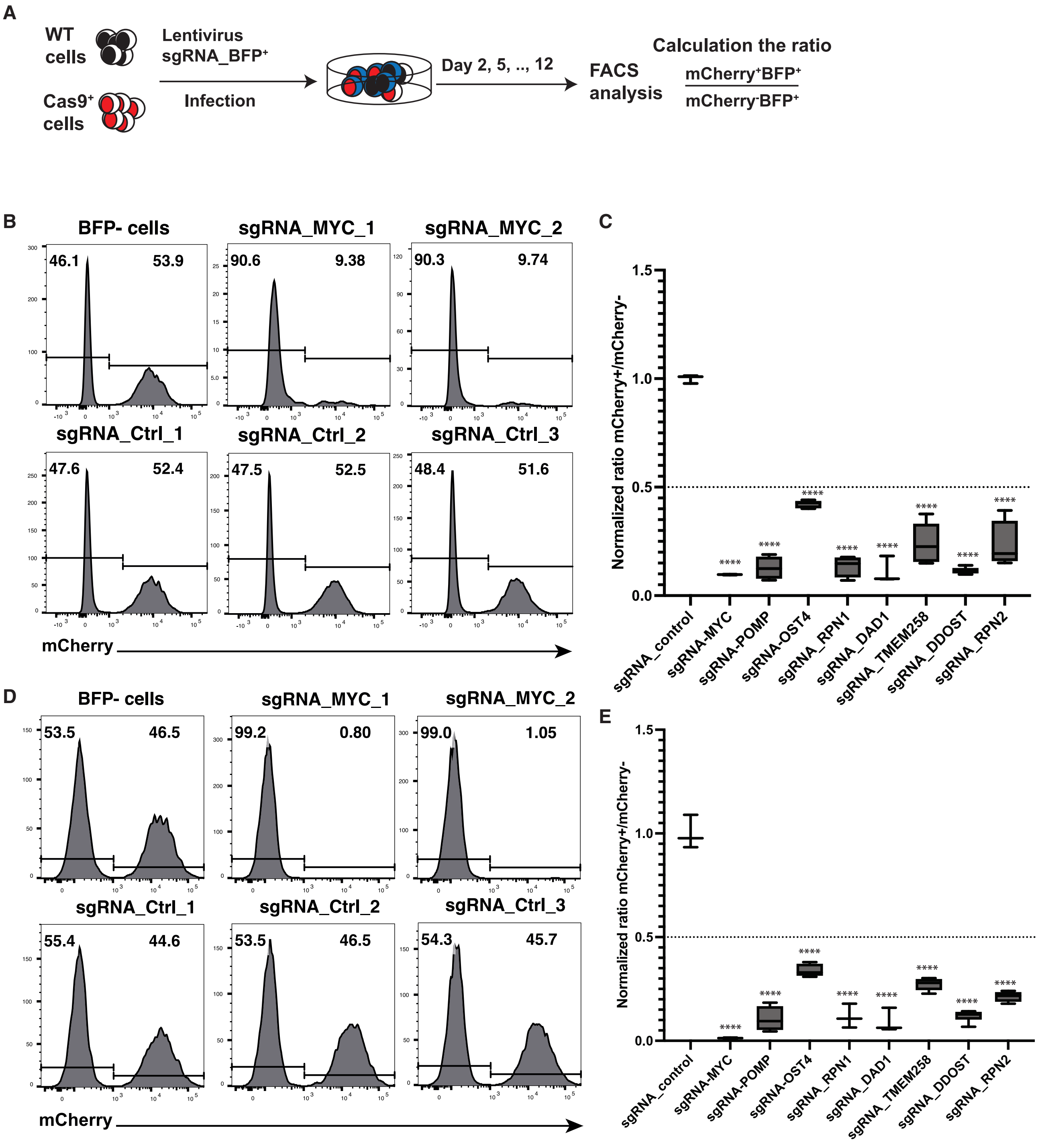

Figure 2. Dual reporter system to assess functions of OST complex's shared subunits in MM cells

(A) Experimental scheme. Wild-type MM cells and Cas9 mCherry expressing MM cells were mixed at 1:1 ratio and then were transduced with lentivirus expressing sgRNAs and BFP. Transduced cells were analyzed by flow cytometry at different timepoints. Flow cytometry data analysis of NCI-H929 (B) and RPMI-8226 (D) cells at day 12 after viral transduction. Pre-gated on BFP^{-} cells, as control, showing the unchanged ratio of double-positive cells and single-positive cells. For other sgRNAs, BFP^{+} cells were pre-gated to identify proportions of mCherry^{+} cells. Histograms showing proportions of mCherry+ cells after being transduced with lentiviruses expressing sgRNAs targeting MYC

(legend continued on next page)

MYC and *POMP* resulted in a significant reduction in knockout cells compared with non-edited cells, thereby confirming the pivotal role of these genes in the survival of MM cells. Notably, the ratio of edited cells to non-edited cells remained stable in the groups targeted with control sgRNAs (Figures 2B–2D and S2). Furthermore, there was no change in the ratio of Cas9-MM cells to wild-type cells in the BFP^{-} cell population (no sgRNA). These findings affirm the reliability of our dual reporter system. Thus, we harnessed this system to evaluate the functions of the six shared subunits of the OST complex in MM cells. We found a pronounced counter-selection of $mCherry^{+}$ cells following the knockout of these subunits (Figures 2C–2E and S2) in a time-dependent manner. Notably, the knockout of *DAD1* and *DDOST* resulted in the most substantial reduction of Cas9-expressing cells. Given the critical role of *DAD1* and *DDOST* in stabilizing the OST complex,[28–30] we focused on characterizing the functions of these two subunits in relation to MM pathology.

Knockout of *DAD1* and *DDOST* exhibited anti-MM effects *in vitro*

To investigate the impact of *DAD1* and *DDOST* knockout in MM cells, we established an inducible knockout system. In brief, MM cells expressing Cas9 were transduced with lentiviruses carrying sgRNAs targeting *DAD1* or *DDOST* under the control of an inducible promoter. Stable cell lines were established following 1 week of selection with puromycin. The addition of doxycycline to the culture medium induced the knockout effect. The induced cells were subsequently subjected to various functional assays including assessments of growth kinetics, cell-cycle progression, and cell apoptosis.

We designed two different sgRNAs that efficiently depleted DAD1 and DDOST proteins. We found that deletion of DAD1 reduced the protein level of DDOST and vice versa (Figures 3A–3C). Our results were in line with a previous publication[29] showing the essential roles of DAD1 and DDOST in stabilizing the OST complexes. We also confirmed that KO of *DAD1* or *DDOST* reduced the expression of catalytic subunits: STT3A and, to a lesser extent, STT3B (Figure S3A). Next, we assessed the effect of *DAD1* or *DDOST*-KO on global N-glycosylation. As lectin binds to all N-glycoproteins, we used Alexa 488-conjugated lectin to assess the global N-glycosylation in KO cells. As expected, the deletion of *DAD1* or *DDOST* robustly reduced the surface and intracellular N-glycoprotein levels (Figures S3B and S3C). Upon the knockout of either *DAD1* or *DDOST*, MM cell growth was severely compromised (Figures 3D–3F). Cell-cycle analyses demonstrated a substantial reduction in the S phase population in knockout cells (Figures 3G–3J). Additionally, we observed an accumulation of sub-G0 phase cells, indicative of apoptotic cells, within the knockout groups (Figures 3K and 3L). Annexin V staining revealed the increase of apoptotic cells in *DAD1* or *DDOST*-KO cells (Figure S3D). Collectively, our findings indicate that the knockout of *DAD1* or *DDOST* exerts a robust anti-MM effect *in vitro* by disrupting the cell cycle and inducing cell death.

Suppression of the OST complex by NGI-1 exhibits anti-MM activity *in vitro*

Previously, a high-throughput screening of compounds from the National Institutes of Health Molecular Libraries Small Molecule Repository identified NGI-1 as a specific inhibitor of the OST complex.[15] In our investigation, we employed this inhibitor to suppress the OST complex in MM cells. In brief, MM cell lines in the logarithmic growth phase were treated with either NGI-1 or DMSO control. Cell samples were collected at different timepoints for subsequent analyses. We tested the sensitivity of both TP53-mutant and TP53-wild-type MM cell lines to NGI-1 and found that MM cells were highly sensitive with NGI-1 regardless of the status of the TP53 gene. The GI 50 (50% growth inhibition index) of NGI-1 in MM cells ranged from 1.2 to 13.8 μM (Figures 4A–4C). At the dose of 10 μM, NGI-1 markedly impeded the growth of several tested MM cell lines (Figure S4A). We next determined whether cell-cycle arrest or cell death was responsible for this observed growth suppression. Cell-cycle analysis revealed that treatment with NGI-1 led to a substantial reduction in S phase cells, with a concomitant accumulation of cells in the G0/G1 phase (Figures 4D, 4E, S4B, and S4C). Intriguingly, prolonged exposure to NGI-1 over a period of 7 days resulted in a notable increase in cell death (Figures 4F, 4G, and S4D). To confirm the activity of NGI-1, we assessed the N-glycosylation of ITGB1, a known N-glycoprotein in MM cells,[31] and found that NGI-1 efficiently abrogated the N-glycosylated forms of ITGB1 in a dose-dependent manner. We also detected the reduction of DAD1, DDOST, STT3A, and STT3B in NGI-1 treated cells (Figure S4E), indicating the suppression of the OST complex. We next sought to investigate the effect of NGI-1 on global N-glycosylation using Alexa 448-conjugated lectin. As a result, we found both global surface and intracellular N-glycoproteins were substantially reduced upon treatment with NGI-1 in a dose-dependent manner (Figures 4H, 4I, S4F, and S4I). To assess the toxicity of NGI-1 on healthy CD34+ cells, we treated these cells with 10 μM NGI-1 that suppressed cell growth of all tested MM cell lines. We found a modest reduction in cell growth, a minor induction of apoptosis, and cell-cycle arrest (Figures S5A–S5C). NGI-1 also caused a minimal apoptosis in human primary fibroblast cells (Figure S5D). In summary, the enzymatic inhibition of the OST complex by NGI-1 detrimentally impacted MM cell growth through a combination of cell-cycle arrest and the induction of apoptotic cell death.

Inhibition of the OST complex sensitizes MM cells to bortezomib treatment

We found that NGI-1 increased the splicing of *XBP1* mRNA in MM cells, indicating the induced endoplasmic reticulum (ER) stress

(sgRNA_MYC_1 and sgRNA_MYC_2) and sgRNAs controls (sgRNA_ctrl_1–3). Graphs summarize the normalized ratio $mCherry^{+}BFP^{+}/mCherry^{-}BFP^{+}$ at day 12 post-transduction with lentiviruses expressing sgRNAs targeting *MYC, POMP,* and other shared subunits of the OST complex in NCI-H929 (C) and RPMI-8226 (E) cells as indicated. Individual flow cytometry data are described in Figure S2. Data are plotted as a mean of normalized ratio calculated from two independent experiments (error bars are the standard deviation) and at least three sgRNAs per gene. ***$p < 0.001$, ****$p < 0.0001$.

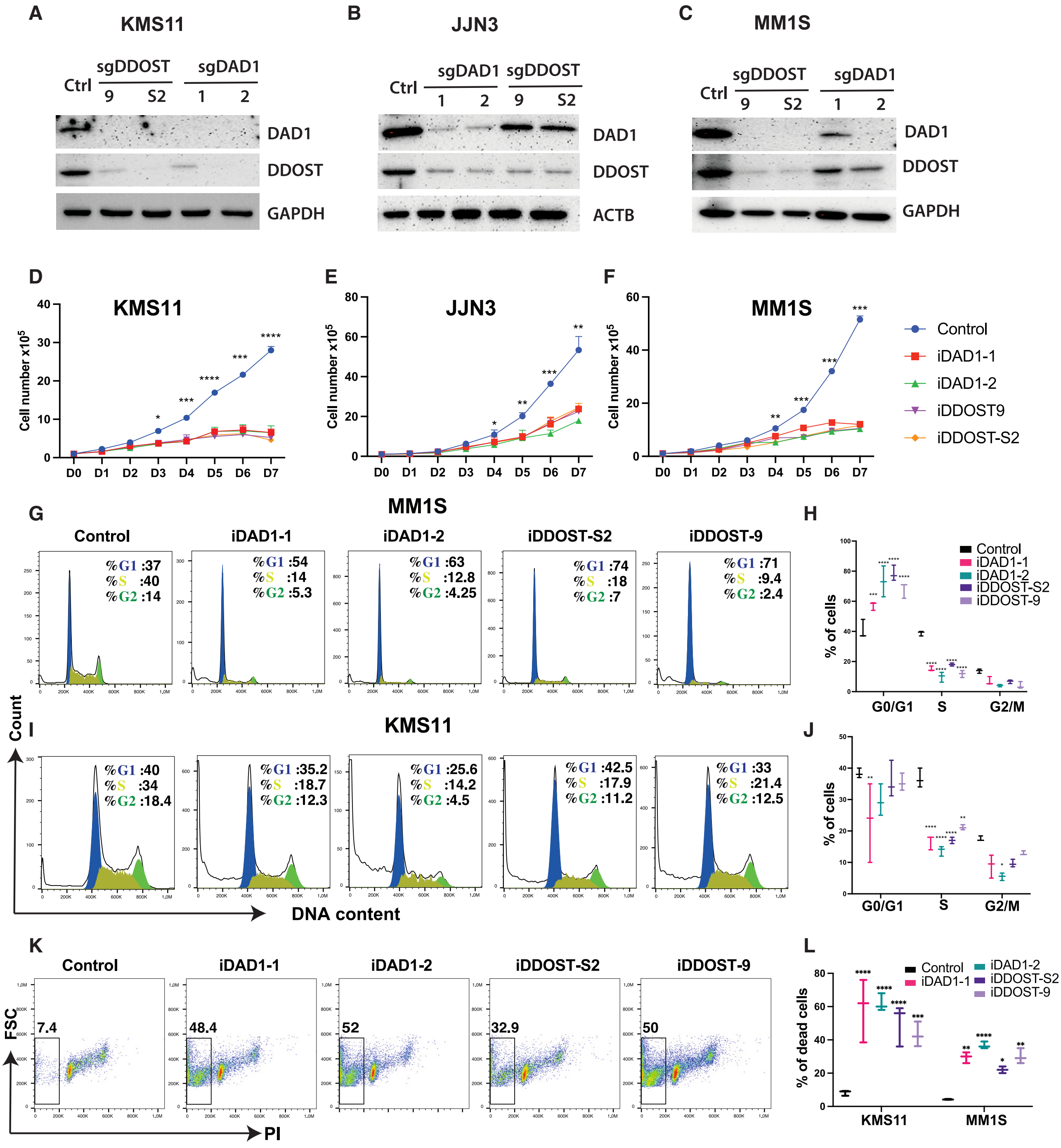

Figure 3. Knockout of *DAD1* and *DDOST* exhibit a strong anti-myeloma *in vitro*

DAD1 and *DDOST* were knocked out in MM cells using two independent sgRNAs each gene: iDAD1-1 and iDAD1-2 for *DAD1*, and iDDOST9 and iDDOST-S2 for *DDOST.* Western blots showing the knockout efficiencies of sgRNAs targeting DAD1 and DDOST in KMS11 (A), JJN3 (B), and MM1S (C). Growth curves of *DAD1* and *DDOST* knockout KMS11 (D), JJN3 (E), and MM1S (F) compared with respective control cells. Data are representative of three independent experiments. Flow cytometry data describe cell-cycle analysis of MM1S (C) and KMS-11 (I) upon knocking out *DAD1* or *DDOST* by two different sgRNAs. Graphs summarize data in (C) and (I) (H and J, respectively) from three independent experiments showing the quantification of cell-cycle phases. (K) Representative flow cytometry data exhibit the accumulation of subG0 phase cells in *DAD1* or *DDOST* knockout MM cells. (L) Graphs summarize the mean of data with standard deviation (error bar) in (K) from three independent experiments in two different indicated cell lines. *$p < 0.05$, **$p < 0.01$, ***$p < 0.001$, and ****$p < 0.0001$.

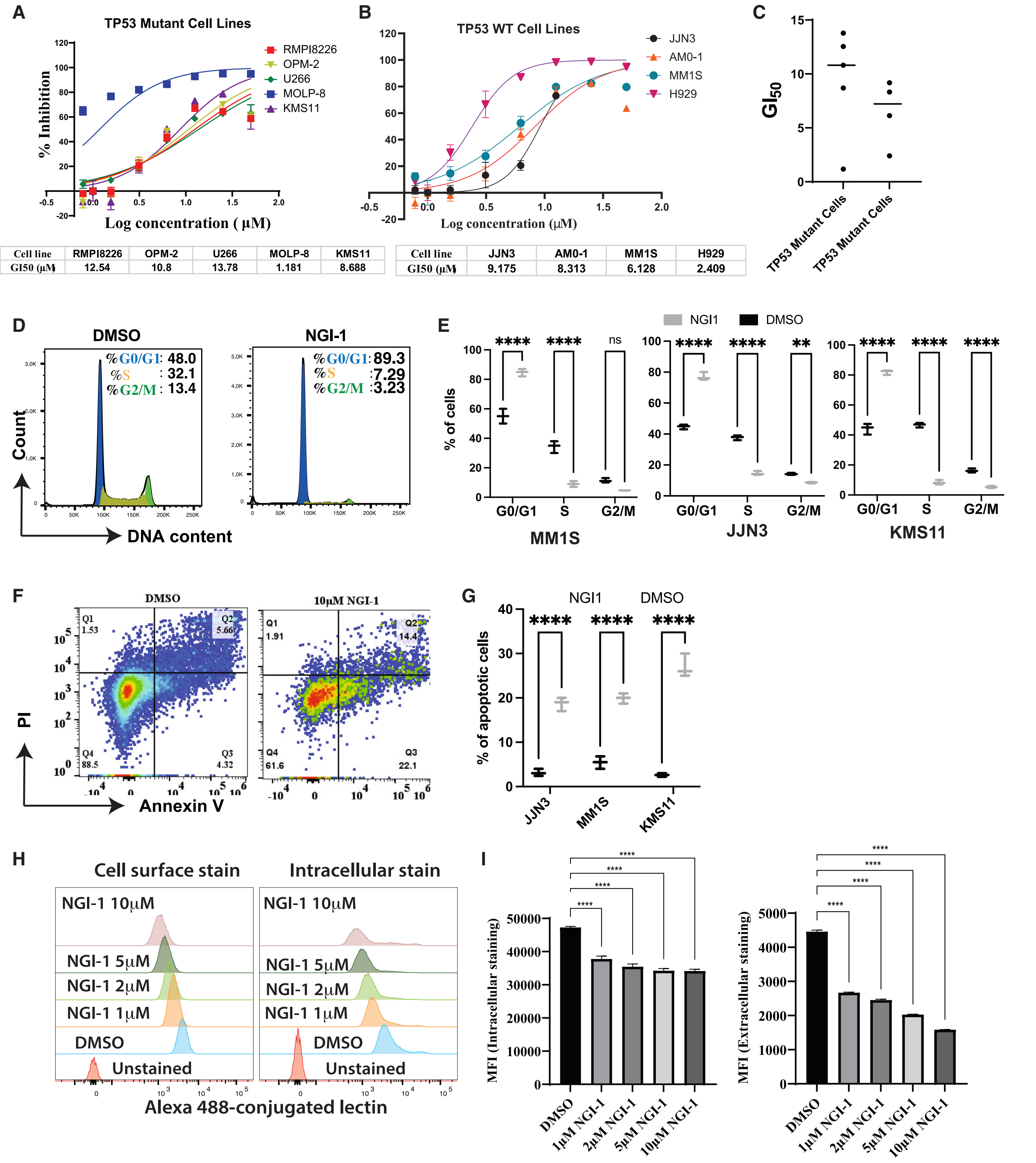

Figure 4. Enzymatic inhibition of the OST complex by NGI-1 suppresses MM cell growth via arresting cell cycle and inducing apoptosis

Dose-dependent response curves of TP53 mutant (A) and TP53 wild-type (WT) MM cell lines upon treating with NGI-1. (C) Graph summarizes the GI_{50} of indicated MM cell lines. (D) Flow cytometry describes cell cycle of MM cell lines upon treatment with NGI-1 10 μM for 3 days. (E) Graphs summarize proportions of cell-cycle phages of three different MM cell lines upon NGI-1 treatment. Data are presented as means with standard deviation (error bar) from three independent experiments. (F) Flow cytometry

(legend continued on next page)

(Figure S6A). Notably, one of the bortezomib's mechanisms of action, a widely used clinical proteasome inhibitor, is to preclude targeted protein degradation that can induce excessive ER stress triggering apoptosis. These findings led us to hypothesize that combining NGI-1 with bortezomib might synergistically enhance the elimination of MM cells. To this end, we treated MM cell lines with NGI-1, bortezomib, or a combination of both agents. We did titration of bortezomib for each cell line to identify a suboptimal dose that minimally impacted MM cell survival. At this tolerated dose, single-agent treatments with either NGI-1 or bortezomib displayed modest effects on MM cell survival. However, the combined treatment of NGI-1 and bortezomib demonstrated a significantly enhanced inhibitory effect (Figures 5A–5D). Consistent with NGI-1 treatment data, we found that *DAD1* or *DDOST*-KO cells were more sensitive to bortezomib treatment than the wild-type cells (Figure S6B).

To evaluate the reproducibility of this combined effect in bortezomib-resistant cells, two bortezomib-resistant MM cell lines (JJN3-BortR and U266-BortR) were generated through prolonged exposure to incrementally increasing bortezomib concentrations. After culturing these cells in a bortezomib-free medium for several weeks, the cells were treated with NGI-1, bortezomib, or combination of the two drugs. Both JJN3-BortR and U266-BortR cell lines exhibited higher resistance to bortezomib compared with their respective parental cell lines (Figure 5E). Notably, the combined treatment of NGI-1 and bortezomib exhibited a robust cytotoxic effect on these bortezomib-resistant MM cell lines (Figures 5F and 5G).

To assess whether this combination effect was additive or synergistic, we employed a luminescent cell viability assay (CellTiter-Glo) to measure ATP production in treated cells. The bortezomib GI 50 values for parental and bortezomib-resistant cell lines were determined. Parental JJN3 and U266 cells exhibited GI 50 values of 2.7 nM and 1.7 nM, respectively, while JJN3-BortR and U266-BortR displayed GI 50 values of 27 nM and 20.4 nM, respectively (Figure 5E).

Next, we treated the cells with NGI-1 alone, bortezomib at the GI 50 dose alone, or a combination of both drugs. The combination treatment significantly suppressed cell viability compared to single-drug treatments (Figures 5H and 5I). Utilizing Synergy Finder,[25] we determined the averaged combination index and found that in all four tested cell lines (parental and bortezomib-resistant cells), the inhibitory effects of the two drugs were indeed synergistic (Figure 5J).

NGI-1 and bortezomib showed synergistic effect to inhibit primary patient MM cells

We next investigated the impact of NGI-1 and bortezomib on primary cells obtained from MM patients. CD138^{+} cells isolated from five newly diagnosed (ND) patients and five relapsed/refractory (RR) patients previously treated with proteosome inhibitors were cultured and treated with NGI-1, different doses of bortezomib, or a combination of NGI-1 and different doses of bortezomib. Cell viability was assessed using a luminescence-based approach after 2 days of drug treatment. NGI-1 alone inhibited cell viability by up to 20% in both ND and RR primary MM cells. As expected, Bortezomib exhibited a dose-dependent cytotoxic effect, particularly in ND patient cells, which displayed high sensitivity even at low bortezomib doses (1–2 nM). Intriguingly, the combination of NGI-1 with tolerated doses of bortezomib efficiently suppressed the viability of ND patient cells. At higher concentrations (4–8 nM), where bortezomib alone approached nearly 100% inhibition, we did not observe a significant additional effect from the combination treatment (Figures 6A and S7A).

For the primary cells isolated from patients pretreated with proteosome inhibitors, we found that these cells were more resistant to bortezomib, as expected. Of note, at the tolerated doses (1–8 nM), addition of NGI-1 robustly reduced the viability of RR patient cells in a bortezomib dose-dependent manner (Figures 6B and S7B). We next determined the bortezomib GI 50 index for individual patient samples and found that, as expected, the RR patient cells exhibited significantly higher bortezomib GI 50 values (5.6 nM) compared with ND patient cells (2.9 nM). Combined treatment of NGI-1 and bortezomib significantly decreased the bortezomib GI 50 for both ND and RR patient cells to 1.1 nM and 3.2 nM, respectively (Figure 6C). To ascertain whether this combined inhibitory effect was additive or synergistic, we calculated the drug-drug combination index using Synergy Finder[32] for each patient sample. Our results indicated that NGI-1 and bortezomib synergistically inhibited the cell viability in primary cells from both ND and RR patients (Figures 6D, S7C, and S7D).

The OST complex regulates the critical pathological pathways of MM cells

To unravel the underlying mechanisms by which the OST complex regulates MM pathology, we investigated global transcriptomic changes in MM cells following treatment with NGI-1. Briefly, MM1S cells were subjected to NGI-1 treatment and harvested at days 2, 3, and 5 for subsequent RNA extraction and sequencing (Figure 7A). Principal component analysis clearly delineated NGI-1-treated cells from their untreated counterparts. Notably, cells treated for 2 days (early response) exhibited cohesive clustering, while those treated for 3 and 5 days (late response) formed another distinct cluster (Figure 7B). Upon NGI-1 treatment, we observed a substantial and time-dependent increase in the number of downregulated genes, up to 7,592 genes, while the number of upregulated genes remained relatively stable, up to 736 genes (Figure 7C). Gene set enrichment analysis (Figures 7D and S8A) unveiled that inhibition of the OST complex downregulated genes enriched in pathways associated with the

describes the Annexin V staining of MM cell lines upon NGI-1 treatment. (G) Graph summarizes the percentage of apoptotic cells in indicated MM cell lines upon NGI-1 treatment. Data are presented as means with standard deviations from three independent experiments. (H) Flow cytometry data showing the surface glycoproteins (left) and intracellular glycoproteins (right) were stained by Alexa 488-conjugated lectin in MM1S cells treated with different concentrations of NGI-1. (I) Graphs summarize the data in (H). Data are presented as mean fluorescence intensity (MFI) from three independent experiments. $^{*}p < 0.05$, $^{**}p < 0.01$, $^{***}p < 0.001$, and $^{****}p < 0.0001$.

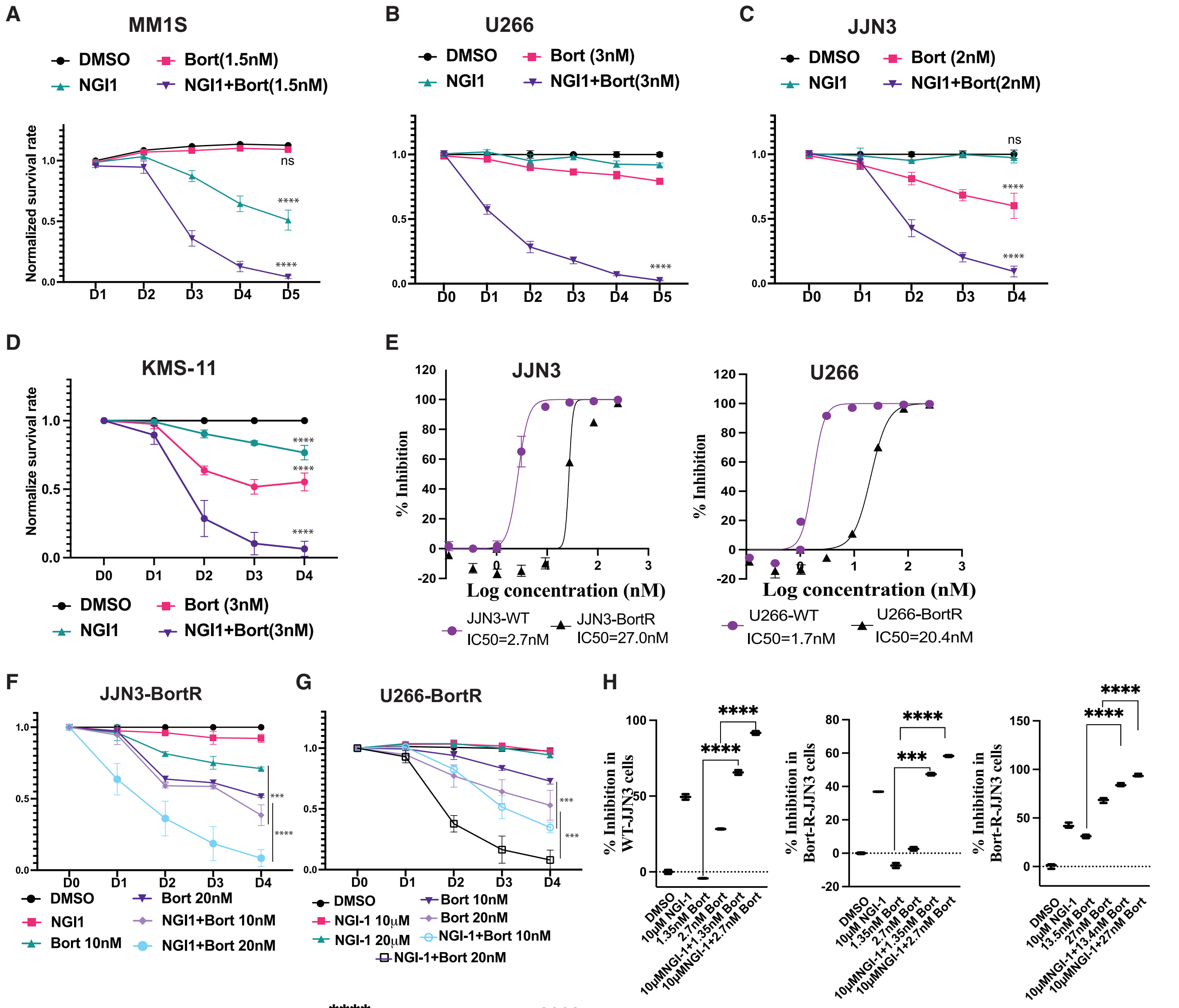

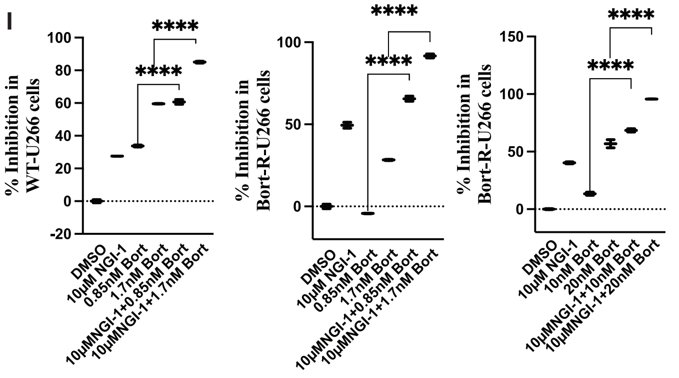

J

Drug-Drug Combination Index by SynergyFinder

Groups	Averaged combination Index	Description
WT-JJN3	24.13 ± 3.42	Synergistic
Bort-R-JJN3	23.96 ± 7.07	Synergistic
WT-U266	15.61 ± 7.09	Synergistic
Bort-R-U266	24.44 ± 1.76	Synergistic

Averaged indexes were calculated from four reference modes. Synergyfinder intepretation: Antagonistic: <-10. Additive: ≥ -10 ≤10. Synergistic: >10.

Figure 5. NGI-1 and bortezomib synergistically kill MM cells *in vitro*

(A–D) MM cell lines were treated with NGI-1 10 μM and indicated suboptimal doses of bortezomib for each cell line. Cell viability was assessed using flow cytometry. Graphs summarize normalized survival data from three independent experiments. (E) JJN3 wild-type (WT), bortezomib-resistant JJN3 (JJN3-BortR), U266 wild-type (WT), and bortezomib-resistant U266 (U266-BortR) were treated with bortezomib at different concentrations to determine the bortezomib dose-dependent curves, bortezomib IC50s

(legend continued on next page)

cell cycle (cell-cycle checkpoints, mitotic spindle, *MYC* target genes, and *E2F* target genes), metabolism (glycolysis and mTORC1 signaling) (Figures 7D, S8C, and S8D), and NF-κB pathways (Figure S8B). On the other hand, NGI-1 treatment upregulated the genes involved in pathways related to apoptosis, inflammatory, and TNF signaling (Figures S8E and S8F). We found the mRNA level of *TNFSF10* encoding for TRAIL, apoptotic inducer in many cancer cells, were significantly induced upon NGI-1 treatment. We checked the protein level of TRAIL in NGI-1 treated cells and found that TRAIL protein was significantly elevated (Figure S8G). These data were consistent with our cell-cycle and apoptosis analyses using cellular models (Figures 3 and 4).

We also performed quantitative mass spectrometry to assess the global protein changes in MM cells treated with NGI-1 for 3 days. We found that upon NGI-1 treatment, 330 proteins significantly downregulated and 252 proteins significantly downregulated compared to control cells (Figures 7E and S9A). Pathway analysis demonstrated a highly overlapping pathways with transcriptomic data. Upon NGI-1 treatment, proteins related to cell-cycle regulatory pathways, MYC target, mTORC1 pathway, and glycolysis were significantly downregulated. We also found proteins related to apoptosis, unfolded protein response, and inflammatory pathways were highly upregulated (Figures 7F and S9B).

The OST complex regulates translation of *MYC* via the mTORC1 pathway

Both transcriptomic and proteomic data showed that the mTORC1 pathway and MYC targets were significantly downregulated upon NGI-1 treatment. We sought to validate these pathways further. We found that suppression of the OST complex by NGI-1 induced the phosphorylation of eIF2α, which occurs in response to ER stress, and reduced the phosphorylation of S6K, indicating the inactivation status of mTORC1 signaling pathway[33] (Figure 7G). The later observation is of interest as mTORC1 is a known upstream regulator of *MYC* mRNA translation.[34,35] KO of *DAD1* or *DDOST* suppressed mTORC1 pathway and induced the phosphorylation of eIF2a (Figure S9D). To further test this idea, we treated MM cells with rapamycin, an mTORC1 inhibitor, and confirmed reduced MYC protein level in MM cells (Figure S9C). Disruption the OST complex by either NGI-1 treatment or deleting *DAD1/DDOST* significantly downregulated MYC protein (Figures 7G and 7H). There was not a significant reduction of *MYC* mRNA in NGI-1-treated cells (Figure S9E). Of note, concurrent treatment of NGI-1 and MG132, another proteosome inhibitor, did not rescue the loss of MYC protein (Figure 7I), suggesting that NGI-1 did not regulate the MYC protein degradation. We evaluated the stability of MYC protein by cycloheximide treatment and found that there was no significant difference in MYC stability under the presence of NGI-1 (Figure 7J).

We next performed polysome profiling to assess the translational regulation of *MYC* upon disrupting the OST complex function. We found that NGI-1 sharply reduced heavy polysomes, indicating reduction in bulk protein synthesis (Figure 7K). Actively translated mRNAs are situated in the heavy polysome fractions. NGI-1 treatment shifted the distribution of *MYC* mRNAs to the lower molecular weight polysome fractions, indicating reduction in *MYC* translation. By comparison, the distribution of *GAPDH* mRNA remained unchanged in response to NGI-1 treatment (Figure 7L). To strengthen this finding, we developed a second approach to assess the translation efficiency of *MYC* mRNA. Cap-dependent translation involves the bindings of translation initiation machinery (eIF4E) on the 5′cap of mRNA.[36] The translation efficiency of an interest mRNA can be measured using RNA immunoprecipitation (RIP) of eIF4E followed by RT-PCR.[37] We adapted this assay and found that eIF4E is abundantly associated with mRNAs of *MYC* and *GAPDH*. Notably, NGI-1 treatment significantly reduced the binding of eIF4E to mRNA of *MYC* but not *GAPDH* (Figure 7M). These data and the polysome profile strongly demonstrated that the OST complex regulates the translation of *MYC*.

Combination treatment of NGI-1 and bortezomib suppressed myeloma cells *in vivo*

Our results strongly support that idea that depletion of the OST complex *in vitro* has potent anti-MM activity. To address whether loss of the OST complex impedes MM tumor growth *in vivo*, we employed a well-established subcutaneous xenograft model. MM cells with stable expression of Cas9 and inducible sgRNAs targeting *DAD1* or *DDOST* were expanded. One day before cell injection, doxycycline was added to induce sgRNA expression. The induced cells were then injected into the flank of NOD Scid Gamma (NSG) mice. Tumor volume was closely monitored until the mice reached terminal criteria (Figure 8A). Knocking out either *DAD1* or *DDOST* robustly suppressed MM tumor growth *in vivo*, resulting in reduced tumor burdens and a significant extension of the survival of transplanted mice across three different MM cell lines (Figures 8B–8E, S10A, and S10B).

Next, we evaluated the *in vivo* effects of NGI-1 and bortezomib using the same xenograft model. JJN3 or JJN3-BortR cells were subcutaneously injected into the flank of NSG mice. Seven days later, we randomized the tumor-bearing mice into different groups for drug treatment: NGI-1 alone (50 mg/kg), bortezomib alone (0.25 mg/kg and 0.5 mg/kg), and a combination of NGI-1 and bortezomib (0.25 mg/kg and 0.5 mg/kg). Tumor progression was closely monitored until reaching termination criteria. Our results indicated that

are depicted. Bortezomib-resistant JJN3 (F) and U266 (G) were treated with with NGI-1 10 μM and indicated suboptimal doses of bortezomib. Cell viability was assessed using flow cytometry. Graphs summarize normalized survival data from three independent experiments. (H and I) Indicated MM cell lines were treated with NGI-1 and indicated doses of bortezomib (IC50s) for 3 days. Viability of treated cells was assessed by Glo-Cell Titer assay. Graphs summarize the mean of data with standard deviation (error bar) from three independent experiments. ***$p < 0.001$, and ****$p < 0.0001$. (J) Drug-drug combination indexes of indicated cell lines upon treatment with NGI-1 and bortezomib were calculated using SynergyFinder software.

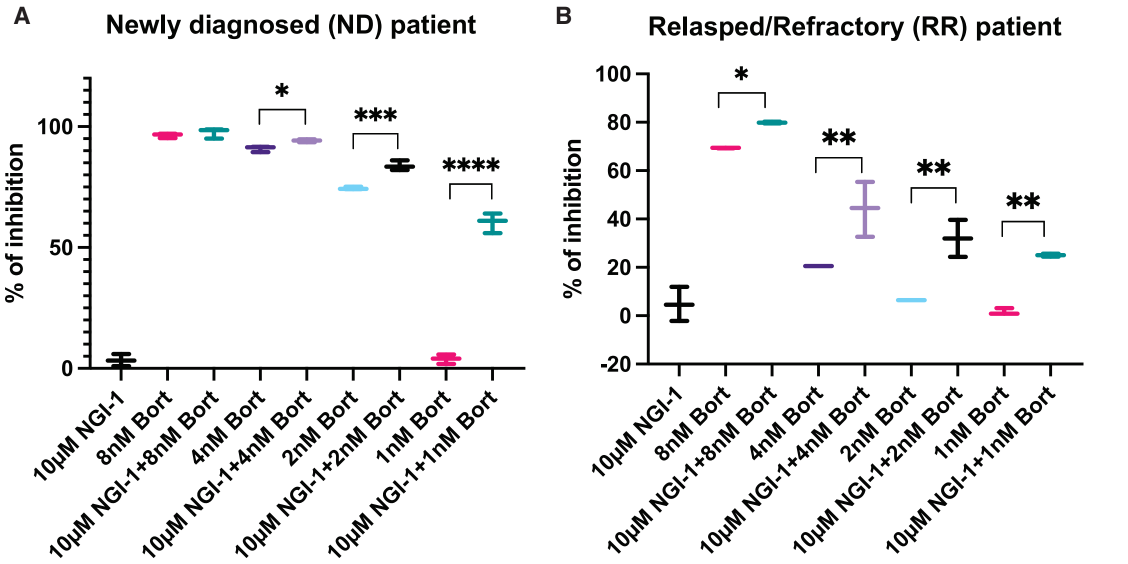

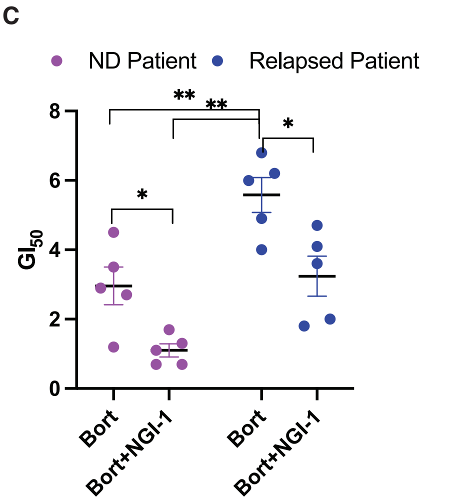

D

Drug-Drug Combination Index by SynergyFinder

Cell Samples	Averaged Combination Index	Description
ND Patient 1	16.95 ± 0.64	Synergistic
ND Patient 2	16.44 ± 3.47	Synergistic
ND Patient 3	19.21 ± 2.67	Synergistic
ND Patient 4	12.72 ± 4.97	Synergistic
ND Patient 5	27.94 ± 0.93	Synergistic
RR Patient 1	21.27 ± 2.13	Synergistic
RR Patient 2	19.13 ± 6.06	Synergistic
RR Patient 3	11.89 ± 0.60	Synergistic
RR Patient 4	11.85 ± 1.14	Synergistic
RR Patient 5	15.69 ± 1.56	Synergistic

Averaged indexes were calculated from four reference modes.
Synergyfinder intepretation: Antagonistic: <-10. Additive: ≥ -10 & ≤10.
Synergistic: >10.

Figure 6. NGI-1 and bortezomib synergistically inhibit primary cells from MM patients

CD138^{+} cells were isolated from representative newly diagnosed (ND) patients (A) and relapsed/refractory (RR) patients (B), treated with NGI-1 and different doses of bortezomib. Cell viability assay was performed after 2 days of treatment. Bar represents the mean of inhibition with standard deviation in individual patient from technical triplicates. (C) GI50s of individual patients (Figures S5A and S5B) were calculated and plotted in the graph. *$p < 0.05$, **$p < 0.01$, ***$p < 0.001$, and ****$p < 0.0001$. (D) table lists the drug-drug combination indexes of individual patients calculated by SynergyFinder.

NGI-1 at a dose of 50 mg/kg exhibited a comparable tumor-suppressing effect in mice groups transplanted with either JJN3 or JJN3-BortR. As expected, bortezomib at doses of 0.25 mg/kg and 0.5 mg/kg efficiently blocked tumor growth in mice transplanted with JJN3 cells. However, single treatments with these doses did not suppress tumor growth in mice transplanted with JJN3-BortR cells. Notably, the combined treatment of NGI-1 and bortezomib (0.25 mg/kg and 0.5 mg/kg) robustly inhibited tumor growth in the mouse group transplanted with JJN3. As bortezomib at a dose of 0.5 mg/kg efficiently suppressed tumor growth, we only observed the combined effect of NGI-1 and bortezomib at the dose of 0.25 mg/kg (Figure 8F). Interestingly, for mice transplanted with JJN3-BortR cells, we observed a pronounced combinational inhibitory effect of NGI-1 and bortezomib (0.5 mg/kg) (Figure 8G).

Next, we validated the combinational treatment in the disseminated xenograft model where human MM cells migrate into the bone marrow. MM1S cells stably expressing luciferase were intravenously injected into irradiated NSG mice. Mice were randomized for drug treatment at day 7 post-implantation (Figure 8I). At week 4 post-transplantation, we analyzed the total bioluminescence index (BLI), human lambda light chain in the peripheral blood, and number of human CD138+ cells in the bone marrow. As expected, bortezomib significantly reduced the total body luminescence (BLI) (Figure 8J)

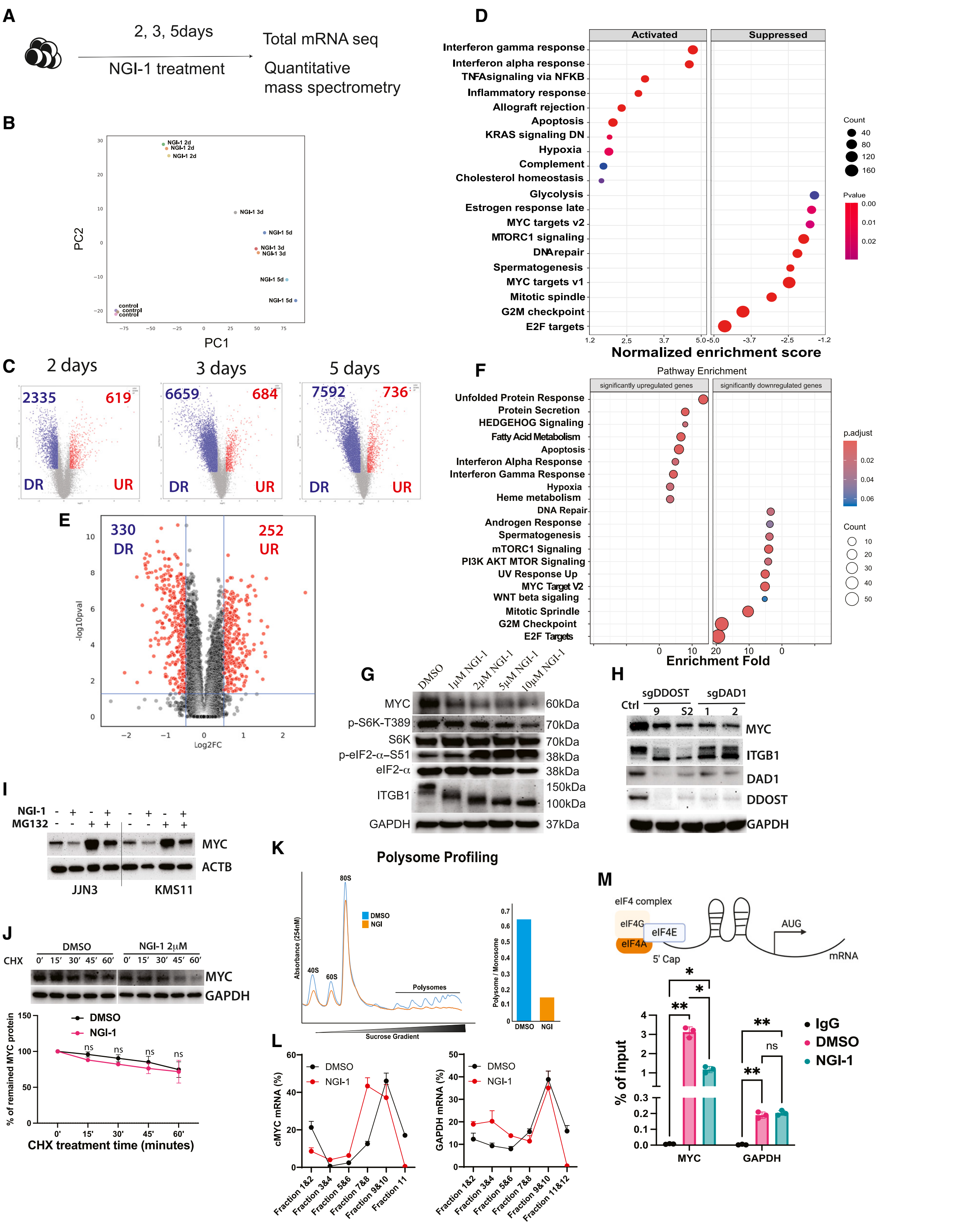

(legend on next page)

and the number of human CD138+ cells in the bone marrow (Figures S10C and S10D) in a dose-dependent manner. Consistent with the subcutaneous model, the combination of NGI-1 and bortezomib robustly suppressed the MM progression in the disseminated model, demonstrated by the reduction of total BLI, the concentration of peripheral blood human lambda light chain, and numbers of human CD138+ cells in the bone marrow (Figures 8I, 8J, 8K, and S10C–S10E). In summary, suppression of the OST complex sensitized MM cells to bortezomib both *in vitro* and *in vivo*.

NGI-1 suppressed the expression of bortezomib-resistant associated genes

Extensive studies have delved into the mechanisms underlying bortezomib resistance.[38] This resistance phenotype has been strongly associated with many factors, including frequent mutations and overexpression of *PSMB5*, a subunit of the proteasome,[39] high activation of the pentose phosphate pathway,[40] elevated levels of *KEAP1/NRF2*,[19,41,42] increased expression of *DDI2* resulting in the activation of *NRF1*,[43] upregulation of Exportin-1 (*XPO1*),[44] *BAX*, and the heat shock protein *HSPB1*.[45] Of note, we found that the suppression of the OST complex by NGI-1 led to a substantial dysregulation in the expression levels of genes related to the bortezomib-resistant phenotype (Figure S11A). Of note, there was overexpression of these gene transcripts in bortezomib-resistant cell lines. Upon NGI-1 treatment, bortezomib-resistant cells exhibited a strong reduction of bortezomib-resistant genes (Figure S11B).

DISCUSSION

Our study is the first to provide preclinical evidence establishing the OST complex as an essential component of MM cells. We found heightened expression of shared OST complex subunits in MM cells, strongly correlating with adverse prognostic outcomes for MM patients. Importantly, genetic or pharmaceutical targeting the OST complex demonstrated potent anti-myeloma activity both *in vitro* and *in vivo*. Notably, previous studies have reported aberrant alterations in serum protein N-glycosylation in MM patients relative to controls.[46,47] The surface N-glycoproteome signature of MM cell lines was previously described[31] and found unique signature of N-glycoprotein in MM cells (membrane-bound, cluster of differentiation, and adherence). De novo N-linked glycosylation sites were detected in the immunoglobulin (Ig) variable regions of lymphoma cells, indicating the pathogenic roles of altered N-linked glycans in B cell lineage malignancies.[48] Recent work by Jutzi et al. underscores the genetic and therapeutic vulnerability of Dpm2-mediated N-glycan synthesis in *CALR*-mutant myeloproliferative neoplasms.[49] The OST complex emerges as a key player in solid tumor progression.[4] Studies by Contessa and colleagues underscore the therapeutic potential of inhibiting the OST complex in receptor tyrosine kinase (RTK)-dependent tumors[15] and EGFR-mutant non-small cell cancer.[14] In the context of normal B cell development, our previous study identified the *Ost4* gene, a subunit of the OST complex, as crucial for mouse B cell survival and differentiation.[16] Other OST complex subunits (*Ostc* and *Stt3a*) and critical enzymes for N-glycosylation (*Dpm1* and *Dpm3*) were indispensable for mouse B cell survival and differentiation into plasma cells.[17] Thus, our findings align with existing evidence, collectively affirming the indispensability of the OST complex in both plasma cells and myeloma cells.

In a subcutaneous xenograft model, we demonstrated that the knockout of either *DAD1* or *DDOST*, two essential shared subunits of the OST complex, effectively suppressed tumor growth *in vivo*, resulting in an extension of the survival of tumor-bearing mice. The pivotal role of *DAD1* and *DDOST* in maintaining OST complex stability has been underscored in the literature.[28–30] Furthermore, our data consistently illustrated that the knockout of *DAD1* or *DDOST* led to a reduction in the protein levels of STT3A and STT3B, the catalytic subunits of the OST complex. Future investigations will be imperative to comprehensively understand the functions of other shared subunits of the OST complex both *in vitro* and *in vivo*.

Intriguingly, our results revealed that the suppression of the OST complex, achieved through the knockout of *DAD1* or *DDOST* or using the specific inhibitor NGI-1, rendered MM cells more sensitive to bortezomib treatment, both *in vitro* and *in vivo*. Bortezomib resistance, attributed to mutations or upregulation of the binding pocket of the β5 subunit (*PSMB5*), leading to impaired drug binding, has been extensively reported.[39,50,51] Additionally, elevated expression of other proteasomal subunits has been identified in bortezomib-resistant cells.[52] MM cell lines resistant to proteasome inhibitors exhibited heightened activity in the serine synthesis pathway (SSP). Notably, the first rate-limiting enzyme in SSP, phosphoglycerate dehydrogenase (*PHGDH*), was significantly elevated in

Figure 7. The OST complex regulates major transcriptomic signatures of MM cells

(A) Experimental scheme. (B) Principal component plots indicate the global transcriptomics of NCI-MM1S cells treated with NGI-1 at different timepoints. (C) Volcano plots describe number of differentially expressed genes in cells treated with NGI-1 compared with control: DR (blue) and UR (red) represent significantly downregulated genes and upregulated genes, respectively. (D) Pathway analysis presents significantly enriched pathways changed upon treatment with NGI-1. (E) Volcano plot showing differentially expressed proteins upon NGI-1 treatment for 3 days in MM1S cells. (F) Pathway analysis for differentially expressed protein revealed the overlapping of transcriptomic and proteomic data. (G) Western blot (WB) data showing the inactivation status of mTOCR1 pathway (phosphorylation of S6K, p-S6K-T389, and eIF2a, *p*-eIF2a-S51) upon NGI-1 treatment in a dose-dependent manner. (H) WB data presenting the decrease in MYC protein level upon deleting *DAD1* (sgDAD1) or *DDOST* (sgDDOST). ITGB1 is a known substrate of the OST complex, lower bands represent the non-glycosylated form of ITGB1. GAPDH was used as a loading control. (I) WB data showing the protein level of MYC in MM cell lines treated with NGI-1 alone, MG132 alone (proteosome inhibitor), or combination of both drugs. (J) WB data showing MYC protein level in control and NGI-1 treated cells pursed with cycloheximide (CHX, 50 μg/mL) (top), graph summarizes data of two independent WBs (bottom). (K) Polysome profile of MM cells treated with DMSO or NGI-1 (left) and quantification of polysome/monosome ratio (right). (L) Graph presenting the distribution of *MYC* mRNA (left) and *GAPDH* mRNA (right) in different polysome fractions. (M) eIF4E-RNA immunoprecipitation followed by real-time PCR. Bar graph summarizes means of percentage of input of *MYC* and *GAPDH* mRNAs associated with eIF4E from three independent experiments. $^{*}p < 0.05$, $^{**}p < 0.01$.

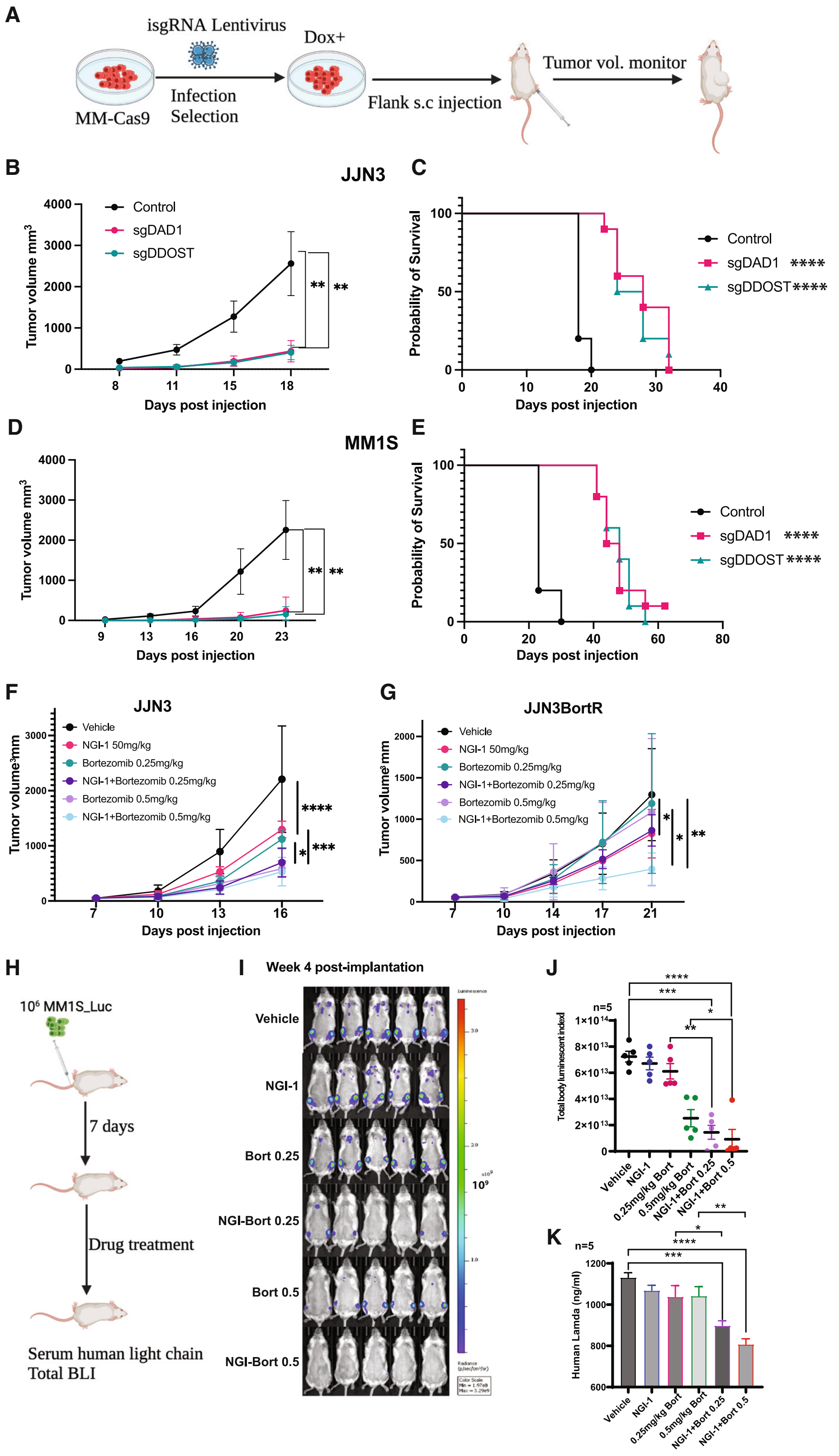

(legend on next page)

CD138$^+$ cells from relapsed MM patients, correlating with inferior survival. Mechanistically, *PHGDH* promotes proliferation and bortezomib resistance by enhancing glutathione synthesis, thereby attenuating bortezomib-induced ROS generation, facilitating effective protein folding, and promoting MM cell survival.[40,53] Strikingly, our study demonstrated a significant reduction in all genes associated with the bortezomib-resistant phenotype (*PSMB5, PHGDH, PSAT1, PSPH, G6PD, KEAP1, DDI2, NRF1, XPO1,* and *BAX*) in cells treated with NGI-1. We also confirmed that these genes were overexpressed in bortezomib-resistant cells and importantly, NGI1 treatment strikingly suppressed these genes in bortezomib-resistant cells. These data provide a rationale for the synergistic anti-MM effect observed with the combined treatment of NGI-1 and bortezomib. Further omics studies comparing parental cells and bortezomib-resistant cells are warranted to identify novel regulators for the bortezomib-resistant phenotype.

Mechanistically, our investigations unveiled that the OST complex regulates the major pathogenic gene signature of MM cells, inducing cell-cycle arrest and TRAIL-mediated apoptosis. Specifically, the OST complex was identified as a key regulator of MYC translation. The reduction of MYC protein levels upon OST complex inhibition led to the lowered expression of MYC target genes crucial for MM cell survival. Moreover, the OST complex was found to modulate the metabolism of MM cells by activating the mTORC1 and glycolysis pathways. Importantly, the highly activated NF-κB pathway in MM,[54] strongly associated with the relapsed/refractory phenotype,[55] was effectively suppressed by NGI-1. Our data are in line with a recent published work showing that the OST complex is required for the activation of the NF-κB pathway.[56] Further studies are warranted to delve into the precise mechanisms through which the OST complex regulates the pathology of MM, including aspects such as bone lesions, homing, migration, and metastasis.

Despite being used in different preclinical models, the global off-targets and potential toxicity of NGI-1 have not been well characterized. The mechanism of how NGI-1 suppresses the OST complex was recently revealed.[56] In this paper, they used a CRISPR base-editor screen to identify NGI-1-resistant variants of STT3A. Using cryo-electron microscopy, they found that NGI-1 binds the catalytic site of STT3A, thus inhibiting the functions of the OST complex. This finding is crucial for developing the next generation of the OST complex inhibitor using a structure-based design. However, how NGI-1 suppresses the OST-B complex is still unknown.

In summary, our study identifies the OST complex as a novel vulnerability in MM treatment. Significantly, we provide compelling preclinical evidence suggesting that the combination of NGI-1 and bortezomib holds promise as a potential therapeutic strategy for proteasome inhibitor-resistant myeloma.

MATERIALS AND METHODS

Cell culture

All cell lines were obtained from on-campus collaborators. MM cell lines NCI-H929, RPMI-8226, KMS11, MM1S, and U266 were cultured in RPMI-1640 medium (Thermo Fisher Scientific, Waltham, MA, USA) supplemented with 10% fetal bovine serum (Corning, USA), 2 mM L-glutamine, sodium pyruvate (Invitrogen), and beta-mercaptoethanol (50 μM, Sigma). Cells were maintained in an incubator with 5% CO_2 at 37°C.

Bortezomib-resistant cell lines (JJN3-BortR and U266-BortR) were generated by continuously cultured JJN3 and U266, respectively, in the presence of increased concentration bortezomib (MedChemExpress, USA). Cells were cultured without bortezomib for several passages prior to experiments.

To generate Cas9 expressing MM cells, we infected MM cells with lentivirus carrying Cas9 and mCherry (Addgene, 70182) as a reporter. mCherry-positive cells were sorted by a BD Fusion sorter (BD, USA). To generate inducible knockout MM cells, Cas9-expressing cells were infected with lentivirus carrying sgRNAs and puromycin-resistant genes (Addgene, 104321). Infected cells were selected in puromycin (0.5–1 μg/mL) -containing medium for at least 1 week. For inducing the expression of sgRNAs, cells were cultured in the medium supplemented with doxycycline (1 μg/mL) into culture medium. Three to 4 days after inductions, cells were harvested for other cellular assays.

All cell lines are tested for mycoplasma periodically. Mycoplasma was tested by PCR approach using the Myco-sniff mycoplasma PCR detection kit according to the manufacturer's manual (MP Biomedicals, USA).

Viability assay for primary MM cells from patients

CD138$^+$ cells from MM patients were kindly provided by the Indiana Myeloma Registry. Cells were seeded on the 96-well plates and treated with NGI-1 and bortezomib as indicated. Two days post-treatment, cell viability was assessed by the CellTiter-Glo luminescence assay (Promega, USA) following the manufacturer's instructions. Percent

Figure 8. Disrupting the OST complex attenuates the MM tumor growth *in vivo*

(A) Experimental scheme. Cas9-expressing cells (MM-Cas9) were infected with lentiviruses expressing sgRNAs (isgRNA) targeting *DAD1* (sgDAD1, combined two sgRNAs) or *DDOST* (sgDDOST, combined two sgRNAs). Dox represents doxycycline. Tumor volumes at different timepoints of mice injected with JJN3 (B) and MM1S (D) were measured and plotted. Data are presented as a mean of tumor volumes of all animals in the experimental groups (n = 5, each sgRNA). Kaplan-Meier curves of mice injected with JJN3 (C) and MM1S (E). Tumor volumes of mice injected with JJN3 (F) or JJN3-BortR (G) cell lines and treated with NGI-1 and bortezomib at the indicated doses. Data are plotted as a mean of tumor volumes of all animals in the experimental groups (n = 5). (H) Experimental scheme for disseminated model. (I) Total bioluminescence index (BLI) pictures of mice treated with indicated condition at week 4 post-implantation. Signal intensity scale is 10^9. (J) Graph summarizes the BLI of transplanted mice (n = 5) treated with different conditions. (K) Graph presents the concentration of human lambda light chain in the peripheral blood collected from treated mice. *$p < 0.05$, **$p < 0.01$, ***$p < 0.001$, ****$p < 0.0001$ (two-way ANOVA test).

inhibition was evaluated by DRC analysis and half maximal inhibitory concentrations (IC50) were calculated using Prism V10 software (GraphPad Software, Inc., LaJolla, CA, USA).

Global N-glycosylation assay

MM cells were treated with DMSO control or NGI-1 at different concentrations. Treated cells were with PBS and stained with Alexa 488-conjugated Concanavalin A lectin (Invitrogen) (100 μg/mL) for 20 min at room temperature. Cells were washed twice with PBS and analyzed by flow cytometry. For intracellular staining, cells were first fixed, permeabilized using a kit from Bio Legend, and then stained with lectin as described above.

Cell-cycle analysis

Cells were washed twice with PBS, fixed with 70% ethanol overnight at 4°C. Fixed cells were washed three times with cold PBS and resuspended into staining buffer containing RNase A (0.5 μg/mL) and propidium iodide (PI, 50 μg/mL). Cells were incubated at 4°C overnight and analyzed by flow cytometry.

Western blot analyses

Cells were harvested, washed with PBS once, and resuspended into RIPA buffer supplemented with proteasome inhibitor cocktail (Millipore). Cells were incubated on ice for 30 min and spun at 15,000 rpm for 30 min at 4°C. Supernatant was collected as whole-cell lysate. Protein was quantified by standard Bradford assay. Ten to 40 μg of proteins was used for western blot following a standard protocol. Antibodies in the study are listed in Table S1.

RNA-seq and data processing

MM1S cells were cultured in the exponential phase and treated with NGI-1 10 μM. Treated cells were harvested after 2, 3, and 5 days of treatment for total RNA extraction using Quick-RNA miniprep kit (Zymo Research, USA). A total of 200 ng of RNA was sent for deep sequencing. Data were processed and analyzed as described previously.[57]

Mass spectrometry

MM1S and JJN3 cells were treated with NGI-1 10 μM for 3 days. Treated cells were harvested for quantitative mass spectrometry. Sample preparation, mass spectrometry analysis, bioinformatics, and data evaluation for quantitative proteomics were performed in collaboration with the Indiana University School of Medicine Center for Proteome Analysis similar to several previously published protocols.[58]

Polysome profiling

Polysome profiling was performed as previously described.[59,60] JJN3 cells were treated with either NGI-1 20 μM or vehicle for 3 days. Cycloheximide was added to each culture dish at a final concentration of 50 μg/mL for 10 min prior to harvesting. Cells were washed with ice-cold PBS solution containing 50 μg/mL cycloheximide and then lysed using 500 μL of cold lysis solution composed of 20 mM Tris·HCl (pH 7.5), 100 mM NaCl, 10 mM MgCl2, 0.4% NP-40, and 50 μg/mL cycloheximide. The lysates were subjected to centrifugation at 15,871 $\times$ *g* for 10 min at 4°C. Subsequently, the cell lysates were layered onto 10%–50% sucrose gradients and ultracentrifuged in a Beckman SW41Ti rotor at 40,000 rpm for 2 h at 4°C. Whole-cell lysate polysome profiles were generated using a piston gradient fractionator (BioComp) and a 254-nm UV monitor with Data Quest Software. Sucrose fractions were collected for RNA extraction. Fractions were combined as indicated. All RNA from fractions were used to synthesize cDNA (first strand cDNA synthesis, NEB). MYC and GADPH mRNAs in different polysome fractions were detected by real-time PCR (QuantStudio 3, Applied Biosystems, USA). Distribution of mRNAs in sucrose fractionation was calculated following the previously described methods.[61,62]

Translation efficiency of mRNA via eIF4E immunoprecipitation coupling with real-time PCR

A total of 10^7 cells were collected and resuspended in 500 μL lysis buffer (50 mM HEPES pH7.5, 140 mM NaCl, 0.5% NP-40, 1 mM EDTA, 0.05% w/v sodium deoxycholate, protease inhibitor, 0.5 mM DTT, 0.5 mM PMSF, and 100 U/mL RNase OUT). Cells were lysed on ice for 2 h. Lysate was cleared by centrifuging at 15,000 rpm for 30 min at 4°C. Cleared lysate was mixed with 10 μg anti-eIF4E (Santa Cruz, sc-271480) and 40 μL Dynabeads Protein G (Invitrogen) and rotated overnight at 4°C. Beads were washed five times with washing buffer (140 mM NaCl, 0.1% NP-40, protease inhibitor, 0.5 mM DTT, 0.5 mM PMSF in PBS). Precipitation was eluted using elution buffer (50 mM HEPES pH 8.0, 2% SDS, 5 mM DTT, and 100 U/mL RNase OUT in water). RNA was extracted using an RNA miniprep kit (Zymo Research) and subjected to cDNA synthesis using an NEB kit. *MYC* and *GADPH* mRNAs were detected by real-time PCR (QuantStudio 3, Applied Biosystems, USA).

Human lambda light chain ELISA

The Nunc 96-well plates (Thermo Scientific) were coated with goat anti-human lambda chain antibody (20 μg/mL) (Southern Biotechnology, #2070-01) in coating buffer (Human Lambda ELISA Kit, Bethyl Laboratories, #E88116) and incubated overnight at 4°C. The plates were then blocked with 5% skim milk in PBS to prevent nonspecific binding, followed by thorough washing. Serum samples were added to the wells and incubated overnight at 4°C. After washing, the plates were incubated with horseradish peroxidase (HRP)-conjugated anti-human IgG (Cell Signaling Technologies, USA) for 2 h at room temperature. After additional washing steps, the plates were developed using the substrate kit, and absorbance was measured at 450 nm. A commercially available standard human lambda chain was used for quantification.

Xenograft model

NOD. Cg-Prkdc^{scid} Il2rg^{tm1Wjl}/SzJ (NSG) mice were bred and maintained in the In Vivo Therapeutics Core (IVT) at Indiana University School of Medicine. All the experiments were performed according to the guidelines of the Institutional Animal Care and Use Committee.

Mycoplasma-negative MM cell lines (5×10^6 cells) were resuspended in 200 μL phosphate-buffered saline (PBS) and injected subcutaneously

into the right flank of NSG mice (6–8 weeks old). Tumor growth was monitored closely with calipers. Tumor-bearing mice were euthanized when tumor volume reached 2,000 mm^3. One week post-transplantation, tumor volumes were measured. Mice were randomized based on tumor volumes into different groups for drug treatments.

For the disseminated model, we generated MM1S cells stably expressing luciferase. A total of 1×10^6 cells were intravenously injected into the sublethally irradiated NSG mice. Seven days after injection, mice were randomized for drug treatment. MM progression was evaluated by total bioluminescence index using IVIS Spectrum CT (PerkinElmer), concentration of human lambda light chain in peripheral blood (ELISA), and numbers of human $CD138^+$ cells in the bone marrow (flow cytometry).

For drug treatment, NGI-1 (50 mg/kg, MedChemExpress) and bortezomib (0.25 and 0.5 mg/kg, MedChemExpress) were injected to the mice intraperitoneally and subcutaneously, respectively.

Statistical analysis

Data are presented as a mean of biological replicates. Error bar is standard deviation. Statistical tests were performed using Prism 10 (GraphPad) using a paired two-tailed Student's t test or two-way ANOVA. ****$p < 0.0001$; ***$p < 0.001$, **$p < 0.01$, *$p < 0.05$.

DATA AVAILABILITY

Data of OST subunits' expression in MM cell lines were extracted from the Cancer Cell Line Encyclopedia (CCLE). Data of MM patients were extracted from the Multiple Myeloma Research Foundation CoMMpass study. RNA-seq data were deposited in the Gene Expression Omnibus database (accession number GSE269271). Raw and processed mass spectrometry data are available via the MassIVE repository, a ProteomeXchange partner (https://massive.ucsd.edu/) with an accession number MSV000095006. All other raw data are available upon request from the corresponding authors.

ACKNOWLEDGMENTS

We thank Dr. Karen Pollok and her staff in the Preclinical Modeling and Therapeutics Core (PMTC) for the help of carrying out the xenograft model. We acknowledge the Flow Cytometry Core facility for assisting with flow cytometry-related works. The Center for Medical Genomics core performed RNA sequencing. We thank the Indiana Myeloma Registry for providing MM patient cells. The Indiana Myeloma Registry is funded in part by support from the Indiana University Precision Health Initiative, Miles for Myeloma, the Harry and Edith Gladstein Chair, and the Omar Barham Fighting Cancer Fund. The mass spectrometry work performed in this work was done by the Indiana University School of Medicine Center for Proteome Analysis. Acquisition of the IUSM Center for Proteome Analysis' instrumentation used for this project was provided by the Indiana University Precision Health Initiative. CCSG P30 provides a discount on all PMTC mice and services (P30CA082709). Support provided by the pilot grant from Indiana University Simon Comprehensive Cancer Center.

The proteomics work was supported, in part, by the Indiana Clinical and Translational Sciences Institute (funded in part by Award Number UL1TR002529 from the National Institutes of Health, National Center for Advancing Translational Sciences, Clinical & Translational Sciences Award) and, in part, by the IU Simon Comprehensive Cancer Center Support Grant (Award Number P30CA082709 from the National Cancer Institute). R.C.W. received support from GM136331.

AUTHOR CONTRIBUTIONS

N.T.T. conceived and developed the project. N.T.T., H.P.N., A.Q.L., M.L., and H.H. designed, performed, analyzed experiments, and interpreted data. E.L. and B.A.W. oversaw and analyzed RNA sequencing data. J.M. and R.C.W. assisted with polysome profiling experiments. S.S. and J.Z. assisted the assessment of mTORC1 pathway. M.A.Z. and R.A. provided myeloma patient cells. R.K. provided materials and commented on the manuscript. N.T.T. and H.P.N. wrote the manuscript.

DECLARATION OF INTERESTS

B.A.W. reports unrelated grants from Bristol Myers Squibb, Genentech, and the Leukemia and Lymphoma Society during the conduct of the study. R.C.W. is a member of the advisory board of HiberCell, Inc.

SUPPLEMENTAL INFORMATION

Supplemental information can be found online at https://doi.org/10.1016/j.omton.2025.200964.

REFERENCES

1. Kyle, R.A., and Rajkumar, S.V. (2004). Multiple myeloma. N. Engl. J. Med. *351*, 1860–1873. https://doi.org/10.1056/NEJMra041875.
2. Palumbo, A., and Anderson, K. (2011). Multiple myeloma. N. Engl. J. Med. *364*, 1046–1060. https://doi.org/10.1056/NEJMra1011442.
3. Kunacheewa, C., and Orlowski, R.Z. (2019). New Drugs in Multiple Myeloma. Annu. Rev. Med. *70*, 521–547. https://doi.org/10.1146/annurev-med-112017-091045.
4. Harada, Y., Ohkawa, Y., Kizuka, Y., and Taniguchi, N. (2019). Oligosaccharyltransferase: A Gatekeeper of Health and Tumor Progression. Int. J. Mol. Sci. *20*, 6074. https://doi.org/10.3390/ijms20236074.
5. Harada, Y., Hirayama, H., and Suzuki, T. (2015). Generation and degradation of free asparagine-linked glycans. Cell. Mol. Life Sci. *72*, 2509–2533. https://doi.org/10.1007/s00018-015-1881-7.
6. Ohtsubo, K., and Marth, J.D. (2006). Glycosylation in cellular mechanisms of health and disease. Cell *126*, 855–867. https://doi.org/10.1016/j.cell.2006.08.019.
7. Dumax-Vorzet, A., Roboti, P., and High, S. (2013). OST4 is a subunit of the mammalian oligosaccharyltransferase required for efficient N-glycosylation. J. Cell Sci. *126*, 2595–2606. https://doi.org/10.1242/jcs.115410.
8. Graham, D.B., Lefkovith, A., Deelen, P., de Klein, N., Varma, M., Boroughs, A., Desch, A.N., Ng, A.C.Y., Guzman, G., Schenone, M., et al. (2016). TMEM258 Is a Component of the Oligosaccharyltransferase Complex Controlling ER Stress and Intestinal Inflammation. Cell Rep. *17*, 2955–2965. https://doi.org/10.1016/j.celrep.2016.11.042.
9. Kelleher, D.J., Karaoglu, D., Mandon, E.C., and Gilmore, R. (2003). Oligosaccharyltransferase isoforms that contain different catalytic STT3 subunits have distinct enzymatic properties. Mol. Cell *12*, 101–111. https://doi.org/10.1016/s1097-2765(03)00243-0.
10. Shibatani, T., David, L.L., McCormack, A.L., Frueh, K., and Skach, W.R. (2005). Proteomic analysis of mammalian oligosaccharyltransferase reveals multiple subcomplexes that contain Sec61, TRAP, and two potential new subunits. Biochemistry *44*, 5982–5992. https://doi.org/10.1021/bi047328f.
11. Braunger, K., Pfeffer, S., Shrimal, S., Gilmore, R., Berninghausen, O., Mandon, E.C., Becker, T., Förster, F., and Beckmann, R. (2018). Structural basis for coupling protein transport and N-glycosylation at the mammalian endoplasmic reticulum. Science *360*, 215–219. https://doi.org/10.1126/science.aar7899.
12. Pfeffer, S., Dudek, J., Gogala, M., Schorr, S., Linxweiler, J., Lang, S., Becker, T., Beckmann, R., Zimmermann, R., and Förster, F. (2014). Structure of the mammalian oligosaccharyl-transferase complex in the native ER protein translocon. Nat. Commun. *5*, 3072. https://doi.org/10.1038/ncomms4072.
13. Ramirez, A.S., Kowal, J., and Locher, K.P. (2019). Cryo-electron microscopy structures of human oligosaccharyltransferase complexes OST-A and OST-B. Science *366*, 1372–1375. https://doi.org/10.1126/science.aaz3505.
14. Lopez Sambrooks, C., Baro, M., Quijano, A., Narayan, A., Cui, W., Greninger, P., Egan, R., Patel, A., Benes, C.H., Saltzman, W.M., and Contessa, J.N. (2018). Oligosaccharyltransferase Inhibition Overcomes Therapeutic Resistance to EGFR Tyrosine Kinase Inhibitors. Cancer Res. *78*, 5094–5106. https://doi.org/10.1158/0008-5472.CAN-18-0505.
15. Lopez-Sambrooks, C., Shrimal, S., Khodier, C., Flaherty, D.P., Rinis, N., Charest, J.C., Gao, N., Zhao, P., Wells, L., Lewis, T.A., et al. (2016). Oligosaccharyltransferase

inhibition induces senescence in RTK-driven tumor cells. Nat. Chem. Biol. *12*, 1023–1030. https://doi.org/10.1038/nchembio.2194.

16. Chu, V.T., Graf, R., Wirtz, T., Weber, T., Favret, J., Li, X., Petsch, K., Tran, N.T., Sieweke, M.H., Berek, C., et al. (2016). Efficient CRISPR-mediated mutagenesis in primary immune cells using CrispRGold and a C57BL/6 Cas9 transgenic mouse line. Proc. Natl. Acad. Sci. USA *113*, 12514–12519. https://doi.org/10.1073/pnas.1613884113.

17. Xiong, E., Popp, O., Salomon, C., Mertins, P., Kocks, C., Rajewsky, K., and Chu, V.T. (2022). A CRISPR/Cas9-mediated screen identifies determinants of early plasma cell differentiation. Front. Immunol. *13*, 1083119. https://doi.org/10.3389/fimmu.2022.1083119.

18. Jovanovic, K.K., Roche-Lestienne, C., Ghobrial, I.M., Facon, T., Quesnel, B., and Manier, S. (2018). Targeting MYC in multiple myeloma. Leukemia *32*, 1295–1306. https://doi.org/10.1038/s41375-018-0036-x.

19. Li, B., Fu, J., Chen, P., Ge, X., Li, Y., Kuiatse, I., Wang, H., Wang, H., Zhang, X., and Orlowski, R.Z. (2015). The Nuclear Factor (Erythroid-derived 2)-like 2 and Proteasome Maturation Protein Axis Mediate Bortezomib Resistance in Multiple Myeloma. J. Biol. Chem. *290*, 29854–29868. https://doi.org/10.1074/jbc.M115.664953.

20. Robak, P., Jarych, D., Mikulski, D., Dróżdż, I., Węgłowska, E., Kotkowska, A., Misiewicz, M., Smolewski, P., Stawiski, K., Fendler, W., et al. (2021). The Prognostic Value of Whole-Blood PSMB5, CXCR4, POMP, and RPL5 mRNA Expression in Patients with Multiple Myeloma Treated with Bortezomib. Cancers *13*, 951. https://doi.org/10.3390/cancers13050951.

21. Driscoll, J.J., Pelluru, D., Lefkimmiatis, K., Fulciniti, M., Prabhala, R.H., Greipp, P.R., Barlogie, B., Tai, Y.T., Anderson, K.C., Shaughnessy, J.D., Jr., et al. (2010). The sumoylation pathway is dysregulated in multiple myeloma and is associated with adverse patient outcome. Blood *115*, 2827–2834. https://doi.org/10.1182/blood-2009-03-211045.

22. Li, C., Wendlandt, E.B., Darbro, B., Xu, H., Thomas, G.S., Tricot, G., Chen, F., Shaughnessy, J.D., Jr., and Zhan, F. (2021). Genetic Analysis of Multiple Myeloma Identifies Cytogenetic Alterations Implicated in Disease Complexity and Progression. Cancers *13*, 517. https://doi.org/10.3390/cancers13030517.

23. Zhan, F., Barlogie, B., Arzoumanian, V., Huang, Y., Williams, D.R., Hollmig, K., Pineda-Roman, M., Tricot, G., van Rhee, F., Zangari, M., et al. (2007). Gene-expression signature of benign monoclonal gammopathy evident in multiple myeloma is linked to good prognosis. Blood *109*, 1692–1700. https://doi.org/10.1182/blood-2006-07-037077.

24. Chng, W.J., Gertz, M.A., Chung, T.H., Van Wier, S., Keats, J.J., Baker, A., Bergsagel, P.L., Carpten, J., and Fonseca, R. (2010). Correlation between array-comparative genomic hybridization-defined genomic gains and losses and survival: identification of 1p31-32 deletion as a prognostic factor in myeloma. Leukemia *24*, 833–842. https://doi.org/10.1038/leu.2010.21.

25. Chng, W.J., Huang, G.F., Chung, T.H., Ng, S.B., Gonzalez-Paz, N., Troska-Price, T., Mulligan, G., Chesi, M., Bergsagel, P.L., and Fonseca, R. (2011). Clinical and biological implications of MYC activation: a common difference between MGUS and newly diagnosed multiple myeloma. Leukemia *25*, 1026–1035. https://doi.org/10.1038/leu.2011.53.

26. Chng, W.J., Kumar, S., Vanwier, S., Ahmann, G., Price-Troska, T., Henderson, K., Chung, T.H., Kim, S., Mulligan, G., Bryant, B., et al. (2007). Molecular dissection of hyperdiploid multiple myeloma by gene expression profiling. Cancer Res. *67*, 2982–2989. https://doi.org/10.1158/0008-5472.CAN-06-4046.

27. Tiedemann, R.E., Zhu, Y.X., Schmidt, J., Yin, H., Shi, C.X., Que, Q., Basu, G., Azorsa, D., Perkins, L.M., Braggio, E., et al. (2010). Kinome-wide RNAi studies in human multiple myeloma identify vulnerable kinase targets, including a lymphoid-restricted kinase, GRK6. Blood *115*, 1594–1604. https://doi.org/10.1182/blood-2009-09-243980.

28. Jones, M.A., Ng, B.G., Bhide, S., Chin, E., Rhodenizer, D., He, P., Losfeld, M.E., He, M., Raymond, K., Berry, G., et al. (2012). DDOST mutations identified by whole-exome sequencing are implicated in congenital disorders of glycosylation. Am. J. Hum. Genet. *90*, 363–368. https://doi.org/10.1016/j.ajhg.2011.12.024.

29. Roboti, P., and High, S. (2012). The oligosaccharyltransferase subunits OST48, DAD1 and KCP2 function as ubiquitous and selective modulators of mammalian N-glycosylation. J. Cell Sci. *125*, 3474–3484. https://doi.org/10.1242/jcs.103952.

30. Sanjay, A., Fu, J., and Kreibich, G. (1998). DAD1 is required for the function and the structural integrity of the oligosaccharyltransferase complex. J. Biol. Chem. *273*, 26094–26099. https://doi.org/10.1074/jbc.273.40.26094.

31. Oldham, R.A.A., Faber, M.L., Keppel, T.R., Buchberger, A.R., Waas, M., Hari, P., Gundry, R.L., and Medin, J.A. (2020). Discovery and validation of surface N-glycoproteins in MM cell lines and patient samples uncovers immunotherapy targets. J. Immunother. Cancer *8*, e000915. https://doi.org/10.1136/jitc-2020-000915.

32. Zheng, S., Wang, W., Aldahdooh, J., Malyutina, A., Shadbahr, T., Tanoli, Z., Pessia, A., and Tang, J. (2022). SynergyFinder Plus: Toward Better Interpretation and Annotation of Drug Combination Screening Datasets. Genom. Proteom. Bioinform. *20*, 587–596. https://doi.org/10.1016/j.gpb.2022.01.004.

33. Wek, R.C. (2018). Role of eIF2alpha Kinases in Translational Control and Adaptation to Cellular Stress. Cold Spring Harb. Perspect. Biol. *10*, a032870. https://doi.org/10.1101/cshperspect.a032870.

34. Csibi, A., Lee, G., Yoon, S.O., Tong, H., Ilter, D., Elia, I., Fendt, S.M., Roberts, T.M., and Blenis, J. (2014). The mTORC1/S6K1 pathway regulates glutamine metabolism through the eIF4B-dependent control of c-Myc translation. Curr. Biol. *24*, 2274–2280. https://doi.org/10.1016/j.cub.2014.08.007.

35. Srivastava, S., Jiang, J., Misra, J., Seim, G., Staschke, K.A., Zhong, M., Zhou, L., Liu, Y., Chen, C., Davé, U., et al. (2022). Asparagine bioavailability regulates the translation of MYC oncogene. Oncogene *41*, 4855–4865. https://doi.org/10.1038/s41388-022-02474-9.

36. Richter, J.D., and Sonenberg, N. (2005). Regulation of cap-dependent translation by eIF4E inhibitory proteins. Nature *433*, 477–480. https://doi.org/10.1038/nature03205.

37. Lucchesi, C., Mohibi, S., and Chen, X. (2021). Measuring Translation Efficiency by RNA Immunoprecipitation of Translation Initiation Factors. Methods Mol. Biol. *2267*, 73–79. https://doi.org/10.1007/978-1-0716-1217-0_5.

38. Kozalak, G., Butun, I., Toyran, E., and Kosar, A. (2023). Review on Bortezomib Resistance in Multiple Myeloma and Potential Role of Emerging Technologies. Pharmaceuticals *16*, 111. https://doi.org/10.3390/ph16010111.

39. Oerlemans, R., Franke, N.E., Assaraf, Y.G., Cloos, J., van Zantwijk, I., Berkers, C.R., Scheffer, G.L., Debipersad, K., Vojtekova, K., Lemos, C., et al. (2008). Molecular basis of bortezomib resistance: proteasome subunit beta5 (PSMB5) gene mutation and overexpression of PSMB5 protein. Blood *112*, 2489–2499. https://doi.org/10.1182/blood-2007-08-104950.

40. Zaal, E.A., Wu, W., Jansen, G., Zweegman, S., Cloos, J., and Berkers, C.R. (2017). Bortezomib resistance in multiple myeloma is associated with increased serine synthesis. Cancer Metab. *5*, 7. https://doi.org/10.1186/s40170-017-0169-9.

41. Barrera, L.N., Rushworth, S.A., Bowles, K.M., and MacEwan, D.J. (2012). Bortezomib induces heme oxygenase-1 expression in multiple myeloma. Cell Cycle *11*, 2248–2252. https://doi.org/10.4161/cc.20343.

42. Weniger, M.A., Rizzatti, E.G., Pérez-Galán, P., Liu, D., Wang, Q., Munson, P.J., Raghavachari, N., White, T., Tweito, M.M., Dunleavy, K., et al. (2011). Treatment-induced oxidative stress and cellular antioxidant capacity determine response to bortezomib in mantle cell lymphoma. Clin. Cancer Res. *17*, 5101–5112. https://doi.org/10.1158/1078-0432.CCR-10-3367.

43. Op, M., Ribeiro, S.T., Chavarria, C., De Gassart, A., Zaffalon, L., and Martinon, F. (2022). The aspartyl protease DDI2 drives adaptation to proteasome inhibition in multiple myeloma. Cell Death Dis. *13*, 475. https://doi.org/10.1038/s41419-022-04925-3.

44. Chanukuppa, V., Paul, D., Taunk, K., Chatterjee, T., Sharma, S., Kumar, S., Santra, M.K., and Rapole, S. (2019). XPO1 is a critical player for bortezomib resistance in multiple myeloma: A quantitative proteomic approach. J. Proteomics *209*, 103504. https://doi.org/10.1016/j.jprot.2019.103504.

45. Li, J., Zhang, X., Shen, J., Guo, J., Wang, X., and Liu, J. (2019). Bortezomib promotes apoptosis of multiple myeloma cells by regulating HSP27. Mol. Med. Rep. *20*, 2410–2418. https://doi.org/10.3892/mmr.2019.10467.

46. Chen, J., Fang, M., Chen, X., Yi, C., Ji, J., Cheng, C., Wang, M., Gu, X., Sun, Q., and Gao, C. (2017). N-glycosylation of serum proteins for the assessment of patients with IgD multiple myeloma. BMC Cancer *17*, 881. https://doi.org/10.1186/s12885-017-3891-3.

47. Zhang, Z., Westhrin, M., Bondt, A., Wuhrer, M., Standal, T., and Holst, S. (2019). Serum protein N-glycosylation changes in multiple myeloma. Biochim. Biophys. Acta. Gen. Subj. *1863*, 960–970. https://doi.org/10.1016/j.bbagen.2019.03.001.

48. Hollander, N., and Haimovich, J. (2017). Altered N-Linked Glycosylation in Follicular Lymphoma and Chronic Lymphocytic Leukemia: Involvement in Pathogenesis and Potential Therapeutic Targeting. Front. Immunol. *8*, 912. https://doi.org/10.3389/fimmu.2017.00912.

49. Jutzi, J.S., Marneth, A.E., Ciboddo, M., Guerra-Moreno, A., Jiménez-Santos, M.J., Kosmidou, A., Dressman, J.W., Liang, H., Hamel, R., Lozano, P., et al. (2022). Whole-genome CRISPR screening identifies N-glycosylation as a genetic and therapeutic vulnerability in CALR-mutant MPNs. Blood *140*, 1291–1304. https://doi.org/10.1182/blood.2022015629.

50. de Wilt, L.H.A.M., Jansen, G., Assaraf, Y.G., van Meerloo, J., Cloos, J., Schimmer, A.D., Chan, E.T., Kirk, C.J., Peters, G.J., and Kruyt, F.A.E. (2012). Proteasome-based mechanisms of intrinsic and acquired bortezomib resistance in non-small cell lung cancer. Biochem. Pharmacol. *83*, 207–217. https://doi.org/10.1016/j.bcp.2011.10.009.

51. Franke, N.E., Niewerth, D., Assaraf, Y.G., van Meerloo, J., Vojtekova, K., van Zantwijk, C.H., Zweegman, S., Chan, E.T., Kirk, C.J., Geerke, D.P., et al. (2012). Impaired bortezomib binding to mutant beta5 subunit of the proteasome is the underlying basis for bortezomib resistance in leukemia cells. Leukemia *26*, 757–768. https://doi.org/10.1038/leu.2011.256.

52. Lawinski, M., Tatarzynka, A., Surowka, E., and Leszczyszyn, J. (1988). [Primary stenosing cholangitis]. Wiad. Lek. *41*, 1675–1678.

53. Wu, X., Xia, J., Zhang, J., Zhu, Y., Wu, Y., Guo, J., Chen, S., Lei, Q., Meng, B., Kuang, C., et al. (2020). Phosphoglycerate dehydrogenase promotes proliferation and bortezomib resistance through increasing reduced glutathione synthesis in multiple myeloma. Br. J. Haematol. *190*, 52–66. https://doi.org/10.1111/bjh.16503.

54. Demchenko, Y.N., Glebov, O.K., Zingone, A., Keats, J.J., Bergsagel, P.L., and Kuehl, W.M. (2010). Classical and/or alternative NF-kappaB pathway activation in multiple myeloma. Blood *115*, 3541–3552. https://doi.org/10.1182/blood-2009-09-243535.

55. Wong, A.H.H., Shin, E.M., Tergaonkar, V., and Chng, W.J. (2020). Targeting NF-kappaB Signaling for Multiple Myeloma. Cancers *12*, 2203. https://doi.org/10.3390/cancers12082203.

56. Lampson, B.L., Ramírez, A.S., Baro, M., He, L., Hegde, M., Koduri, V., Pfaff, J.L., Hanna, R.E., Kowal, J., Shirole, N.H., et al. (2024). Positive selection CRISPR screens reveal a druggable pocket in an oligosaccharyltransferase required for inflammatory signaling to NF-κB. Cell. *187*, 2209–2223.e16. https://doi.org/10.1016/j.cell.2024.03.022.

57. Nguyen, H.P., Le, A.Q., Liu, E., Cesarano, A., DiMeo, F., Perna, F., Kapur, R., Walker, B.A., and Tran, N.T. (2023). Protein arginine methyltransferase 1 is a therapeutic vulnerability in multiple myeloma. Front. Immunol. *14*, 1239614. https://doi.org/10.3389/fimmu.2023.1239614.

58. Grecco, G.G., Munoz, B., Di Prisco, G.V., Doud, E.H., Fritz, B.M., Maulucci, D., Gao, Y., Mosley, A.L., Baucum, A.J., and Atwood, B.K. (2022). Prenatal Opioid Exposure Impairs Endocannabinoid and Glutamate Transmission in the Dorsal Striatum. eNeuro *9*. ENEURO.0119-22.2022. https://doi.org/10.1523/ENEURO.0119-22.2022.

59. Holmes, M.J., Misra, J., and Wek, R.C. (2022). Analysis of Translational Control in the Integrated Stress Response by Polysome Profiling. Methods Mol. Biol. *2428*, 157–171. https://doi.org/10.1007/978-1-0716-1975-9_10.

60. Teske, B.F., Baird, T.D., and Wek, R.C. (2011). Methods for analyzing eIF2 kinases and translational control in the unfolded protein response. Methods Enzymol. *490*, 333–356. https://doi.org/10.1016/B978-0-12-385114-7.00019-2.

61. Poria, D.K., and Ray, P.S. (2017). Polysome Analysis. Bio. Protoc. *7*, e2192. https://doi.org/10.21769/BioProtoc.2192.

62. Tran, N.T., Su, H., Khodadadi-Jamayran, A., Lin, S., Zhang, L., Zhou, D., Pawlik, K.M., Townes, T.M., Chen, Y., Mulloy, J.C., and Zhao, X. (2016). The AS-RBM15 lncRNA enhances RBM15 protein translation during megakaryocyte differentiation. EMBO Rep. *17*, 887–900. https://doi.org/10.15252/embr.201541970.

OMTON, Volume *33*

Supplemental information

The oligosaccharyltransferase complex is an essential component of multiple myeloma plasma cells

Hong Phuong Nguyen, Enze Liu, Anh Quynh Le, Mahesh Lamsal, Jagannath Misra, Sankalp Srivastava, Harikrishnan Hemavathy, Reuben Kapur, Mohammad Abu Zaid, Rafat Abonour, Ji Zhang, Ronald C. Wek, Brian A. Walker, and Ngoc Tung Tran

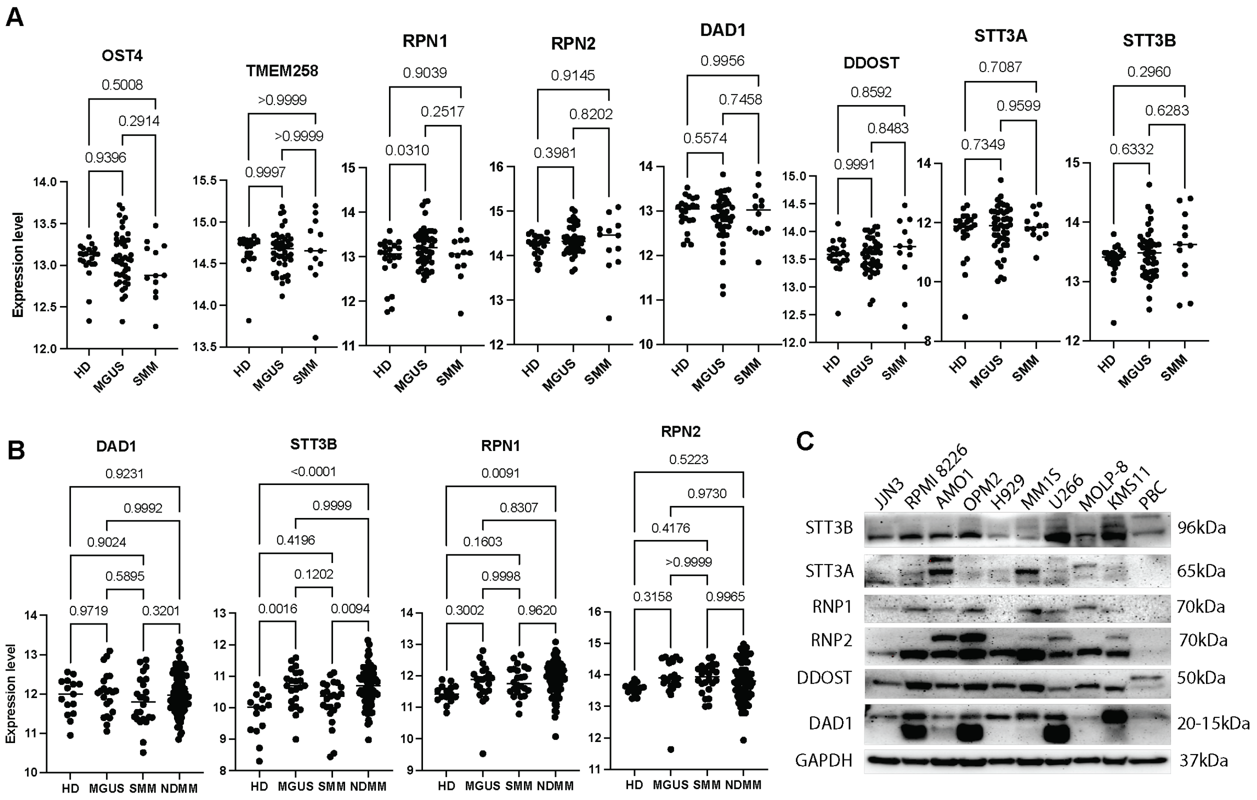

Figure S1. Expression level of OST's subunits in plasma cells isolated from healthy donor (HD), Monoclonal Gammopathy of Undetermined Significance (MGUS) patients, and Smoldering Multiple Myeloma patient (SMM). Data were extracted from microarray datasets: GSE5900 (A) and GSE6477 (B). (C) Western blot showing the protein levels of OST complex in different multiple myeloma cell lines and healthy peripheral blood cells.

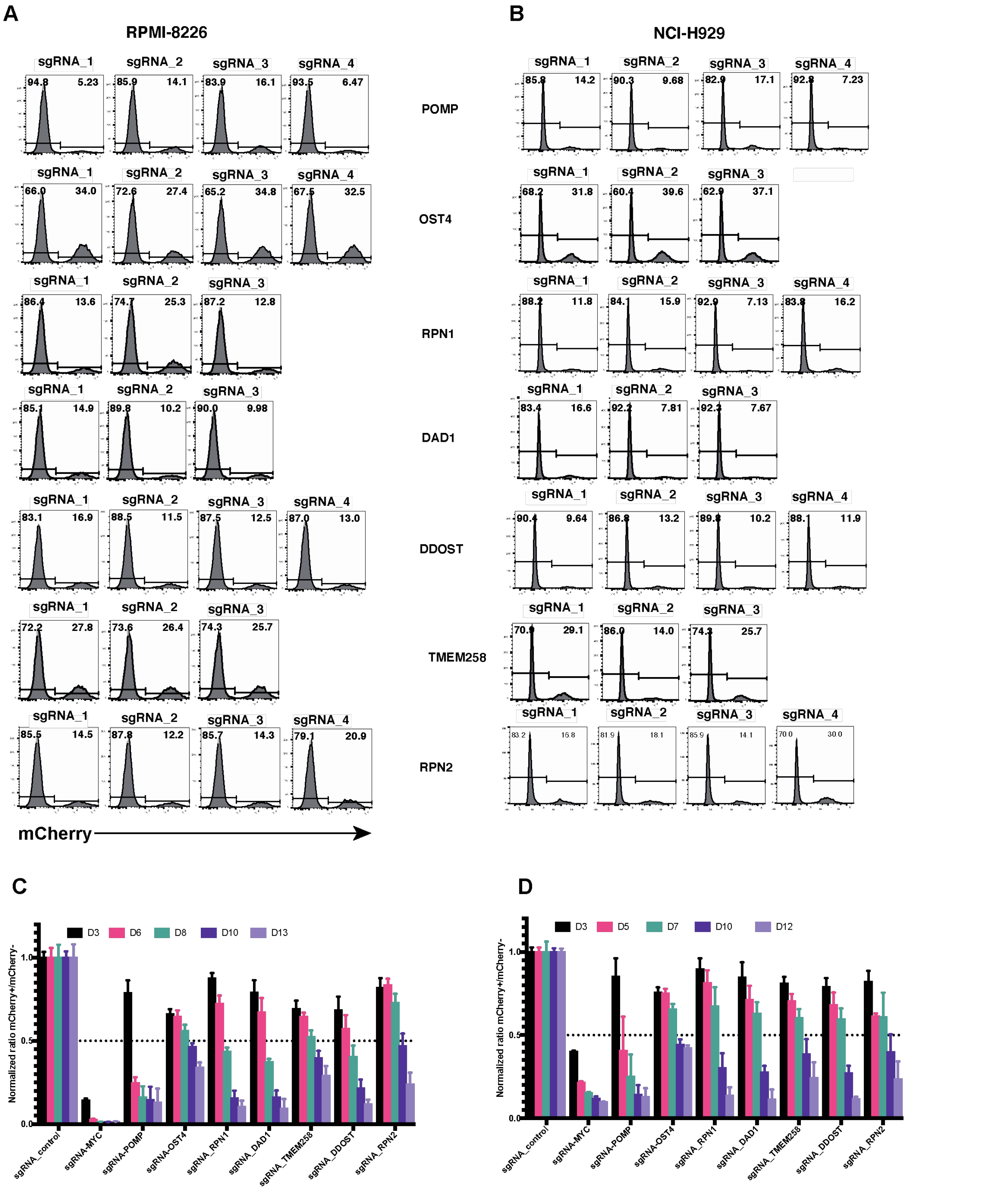

Figure S2. Histograms showing the proportion of mCherry+ cells (Cas9) in pre-gated BFP+ cells at day 12 after transducing with lentiviruses expressing indicated sgRNAs. Data from two cell lines were presented: RPMI-8226 (A) and NCI-H929 (B). Bar graphs summarize the normalized ratio of mCherry+/mCherry- in the BFP+ cells at different time points. Two cell lines were used in this experiment: RPMI-8226 (C) and NCI-H929 (D).

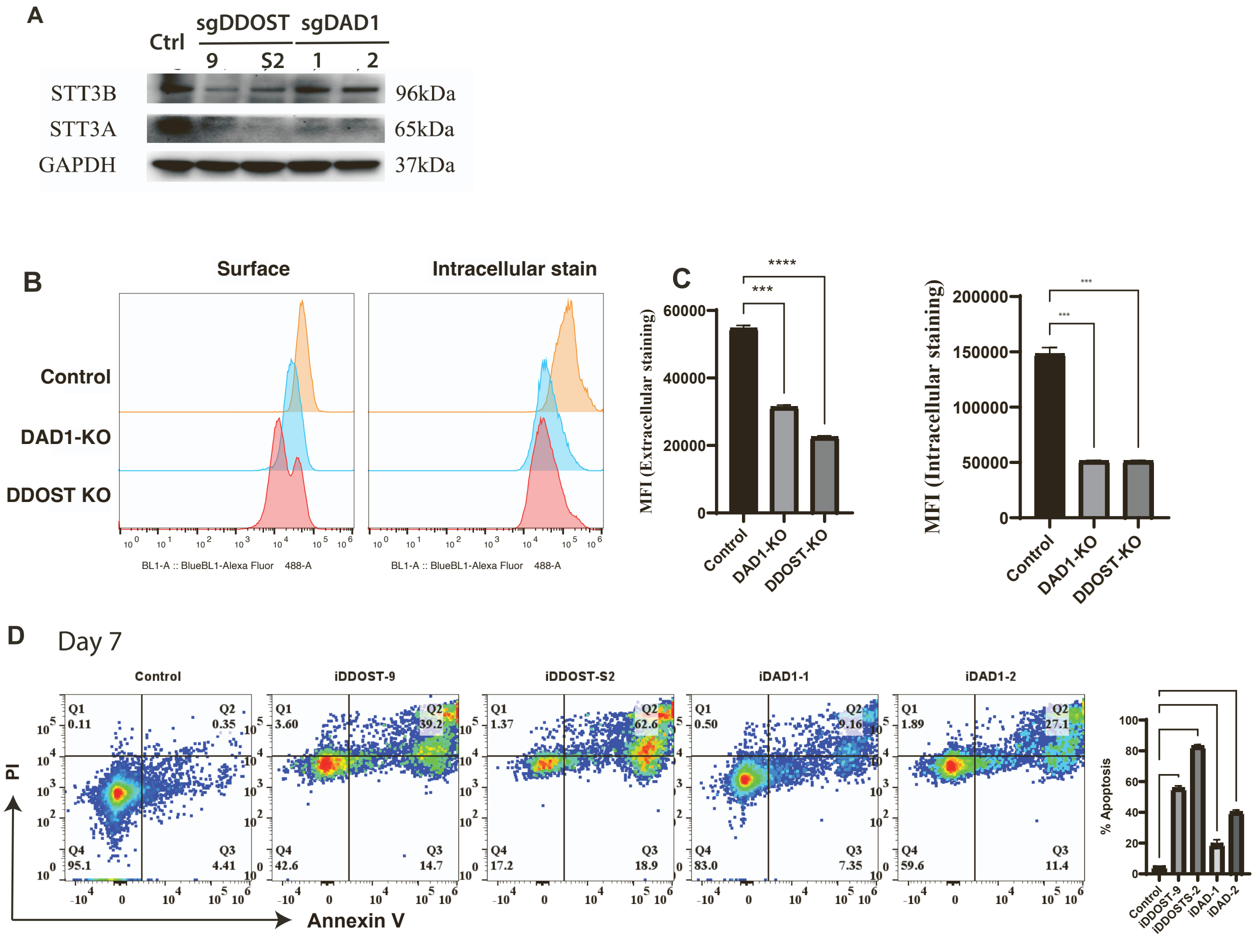

Figure S3. (A) Western blot showing the protein level of STT3A and STT3B in DDOST or DAD1-KO myeloma cells. We designed two sgRNAs per gene. (B) Histogram presenting the global surface and intracellular glycoproteins in DAD1 or DDOST-KO cells. (C) Data were presented as mean of fluorescent intensity (MFI). (D) Flow cytometry data showing the apoptosis of DAD1 or DDOST-KO cells using Annexin V staining (left). Data were summarized in the bar graph as means of percentage of apoptotic cells. *p<0.05, **p<0.01, ***p<0.001, and ****p<0.0001.

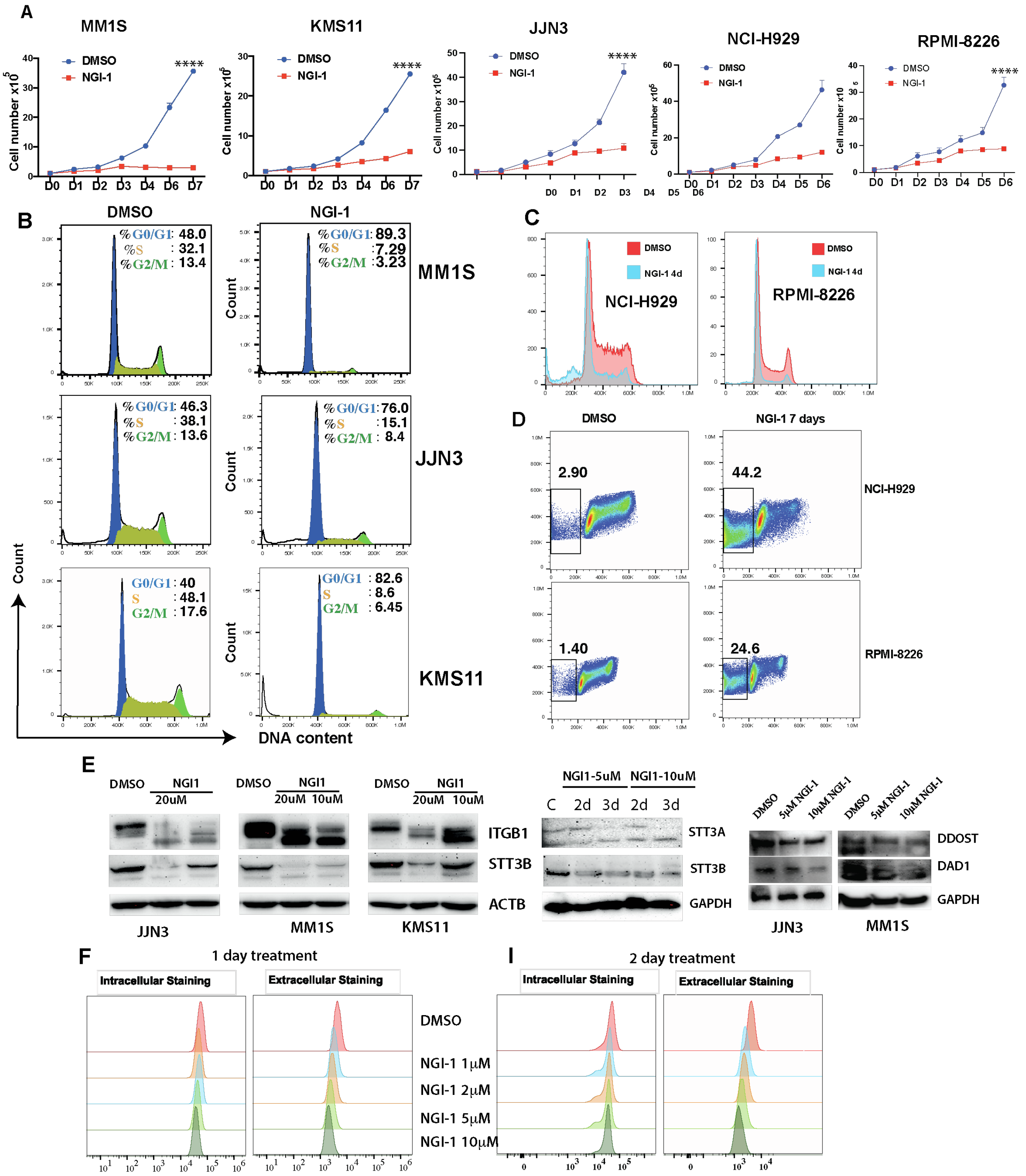

Figure S4. Dose-dependent curves presenting the sensitivity of TP53 mutant (A) and TP53 wildtype MM cell lines to NGI-1 treatment. NGI-1 GI50 (growth inhibition index) of these cell lines were calculated and presented in (C). Growth curves (D), cell cycle profiles (E), and cell death analysis (F) of NCI-H929 and RPMI-8226 upon being treated with DMSO or NGI-1 10mM. (G) western blot data from 3 different MM cell lines upon being treated with NGI-1. ITGB1 is known glycoprotein in MM cells, STT3A and B are catalytic subunit of the OST complex. GAPDH and Actin B (ACTB) are internal control. (H) Alexa-488-conjugated lectin staining for MM1S cells treated with different concentrations of NGI-1 for 2 days. (I) Flow cytometry data

showing the lectin staining for control and DAD1 or DDOST KO cells. cells were analyzed by flow cytometry to detect global surface (left) and intracellular (right) N-glycoproteins. (Growth curve (J), apoptosis (K), and cell cycle profiles (L) of peripheral blood CD34+ cells upon treated with NGI-1

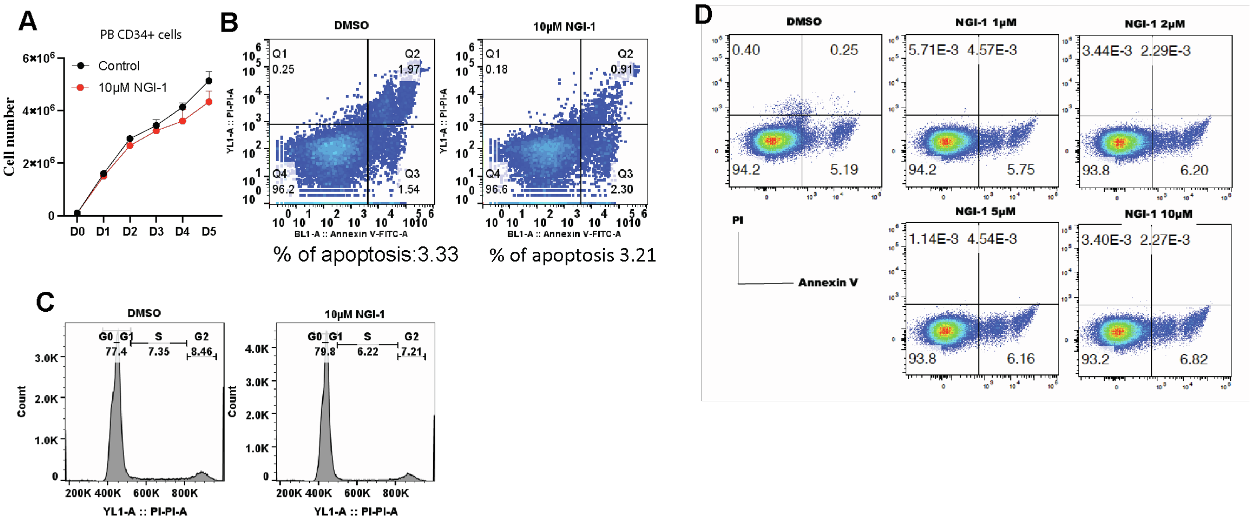

Figure S5. Growth curves (A), apoptosis (B), and cell cycle profile of human CD34+ cells upon treating with NGI-1 or DMSO control. (D) Apoptosis profile of human primary fibroblast cells upon treated with different doses of NGI-1 for 3 days.

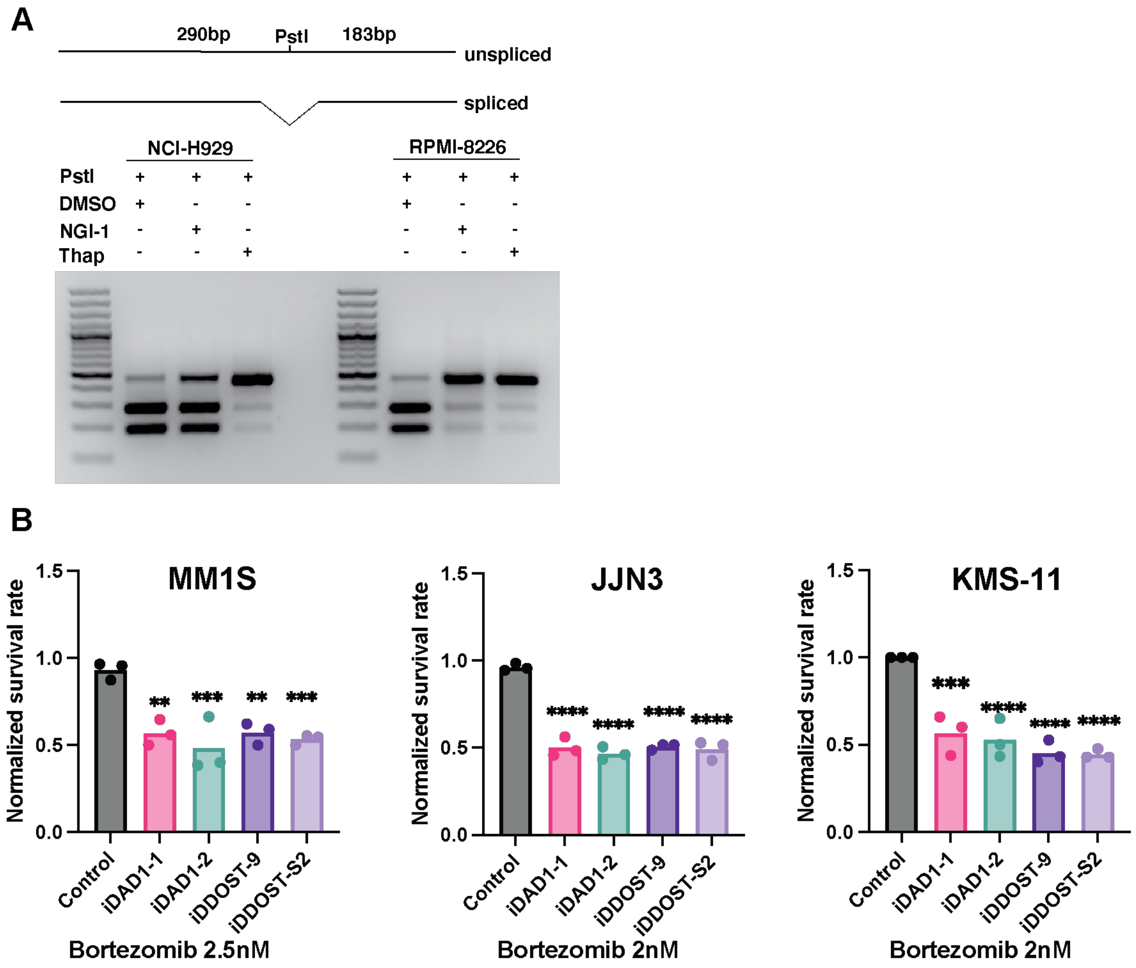

Figure S6. (A) XBP1 splicing assay indicating the ER stress status of MM cell lines treated with NGI-1. PstI is restriction enzyme site located in the XBP1 unspliced transcript. Upon ER stress induction, XBP1 will be spliced and removed the PstI site. Thapsigagin servers as positive control for ER stress inducer. Agarose gel showing the PCR products after digesting with PstI. (B) Bar graphs presenting the normalized survival of MM cell lines upon knocking out of DAD1 or DDOST in the presence of indicated bortezomib doses.

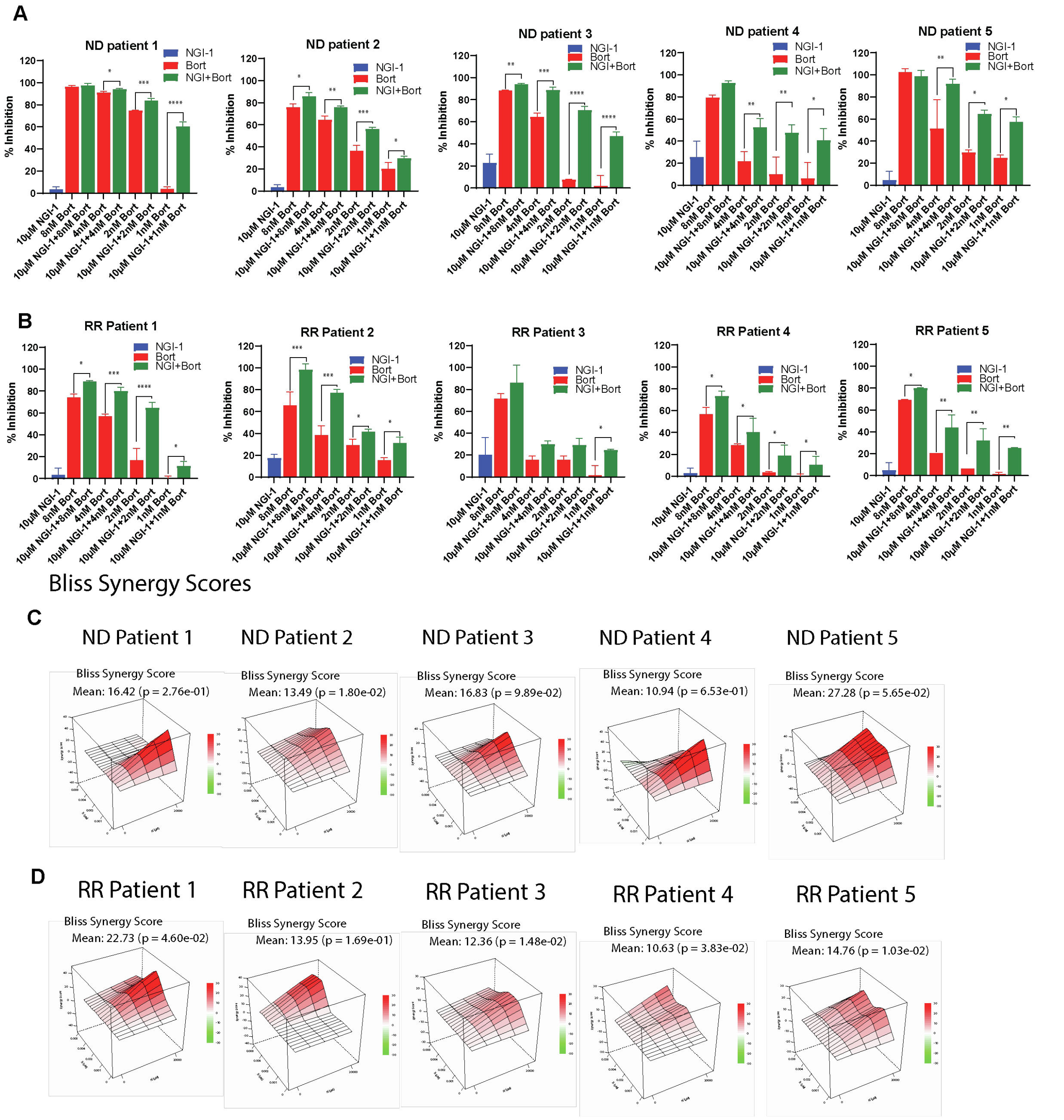

Figure S7. Bar graphs summarize the cell viability assay of newly diagnosed (ND) patients (A) and relapsed/refractory (RR) patients (B) upon being treated with NGI-1 and different indicated doses of bortezomib. Data from each patient is the mean of technical tripreplicates. *p<0.05, **p<0.01, ***p<0.001, ****p<0.0001. Graphs presenting the means of Bliss Synergy scores in each ND (C) or RR (D) patients upon being treated with NGI-1 and bortezomib.

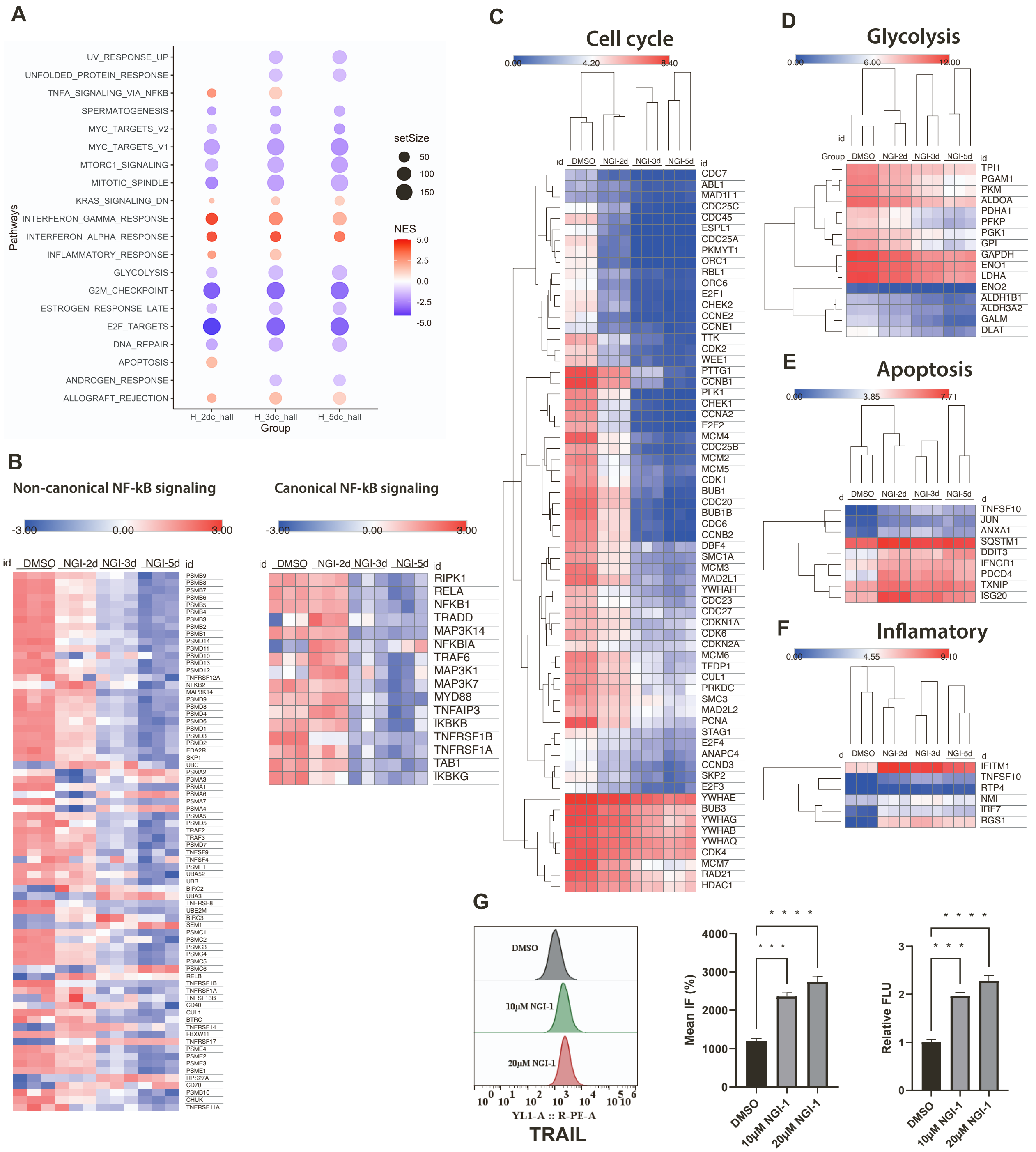

Figure S8. (A) pathway analysis of differential expressed genes in MM1S cell upon being treated with NGI-1 for different days. Heatmap presenting the differential expressed genes in NF-kB signaling pathways (B), Cell cycle (C), Glycolysis (D), Apoptosis (E), and Inflammatory (F) upon being treated with NGI-1. (G) Flow cytometry (left) data depicts the expression of TRAIL in MM cells treated with NGI-1 at different doses for 3 days. Bar graphs (right) summarize the mean fluorescent intensity and relative fluorescent units calculated from C.

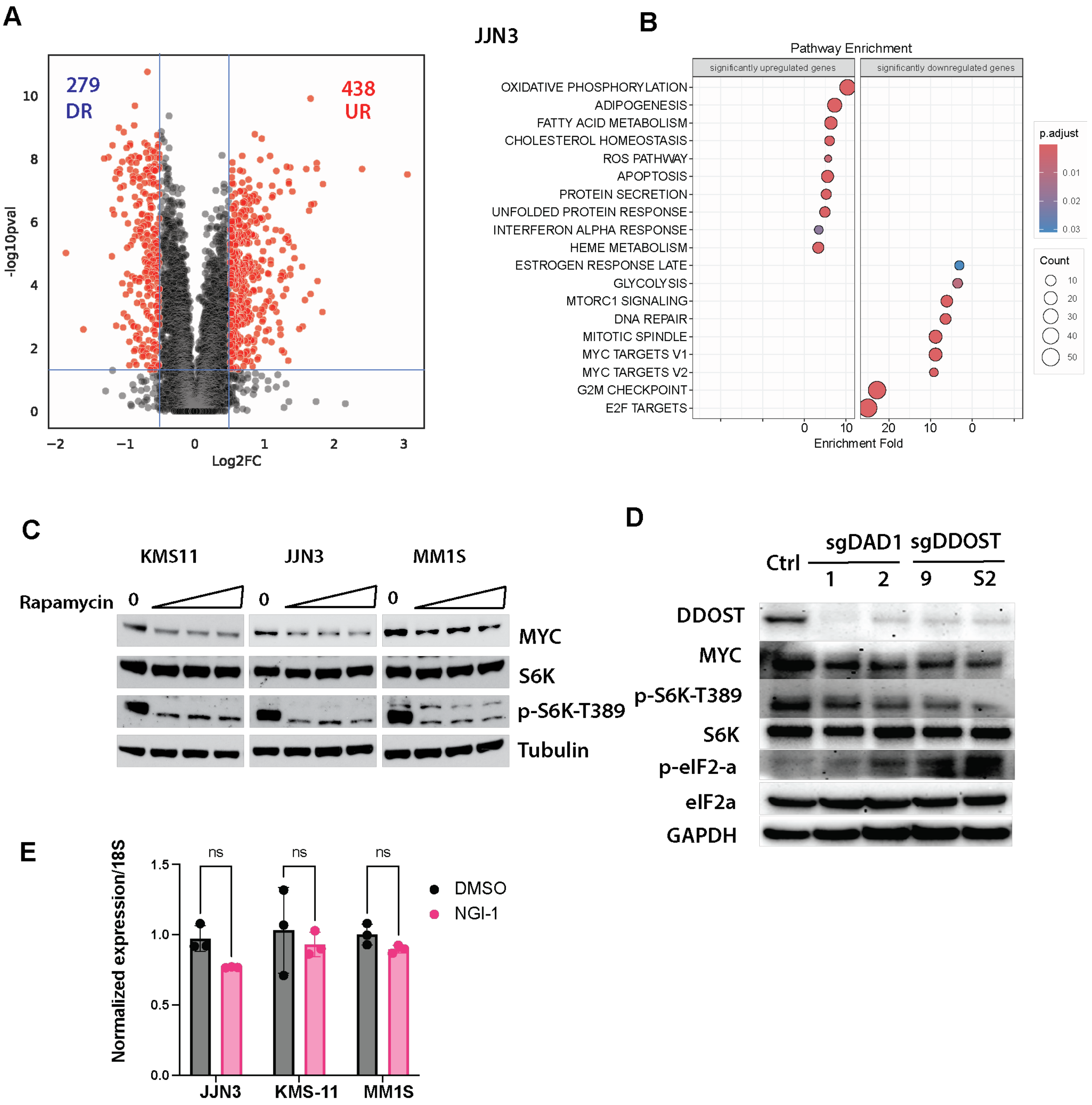

Figure S9. (A) Volcano plot showing differentially expressed proteins (DEP) in JJN3 cells upon treated with NGI-1, DR (downregulated), UR (upregulated). (B) Pathway analysis of DEPs. (C) Western blot showing the activity of mTOCR1 pathway and MYC upon treated with rapamycin (50nM, 100nM and 200nM) at different concentrations. Tubulin was used as a loading control. (D) Western blot showing the protein level of MYC and activity of the mTOCR1 pathway in DAD1 or DDOST-KO myeloma cells. (E) mRNA level of MYC in MM cells upon treating with NGI-1 10μM.

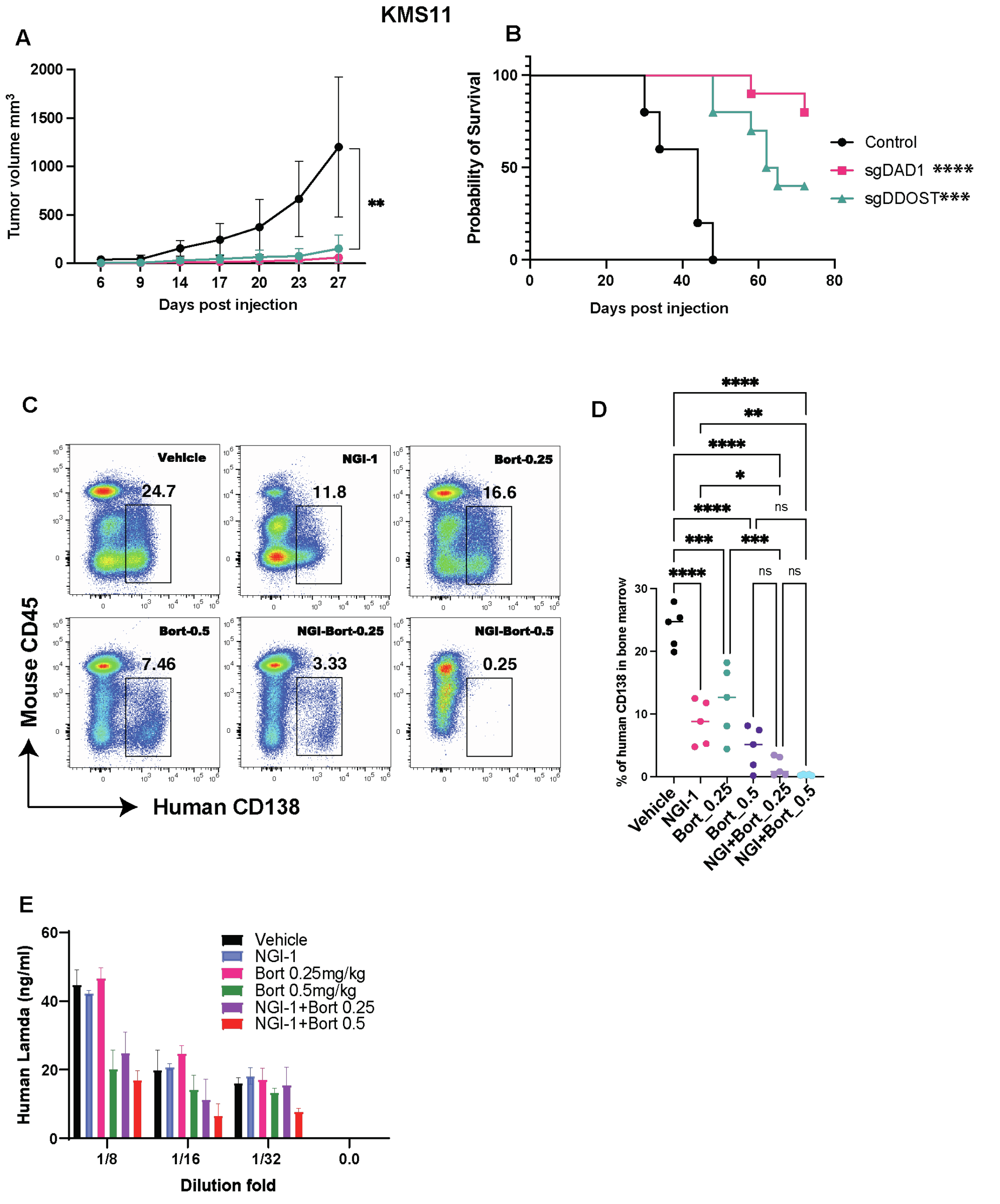

Figure S10. (A) Tumor volumes and Kaplan survival curves of mice subcutaneously implanted with KMS11 control or DAD1/DDOST KO cells. (C) Flow cytometry presenting the percentages of human CD138+ cells in the bone marrow of mice intravenously injected with MM1S cells after different treatment conditions. (D) Graph summarizes data in C from different drug treatment groups (N=5). (E) ELISA showing the level of human lambda levels in peripheral blood of mice treated with different conditions (different dilution of serum) at termination of experiment (4 weeks post-implantation).

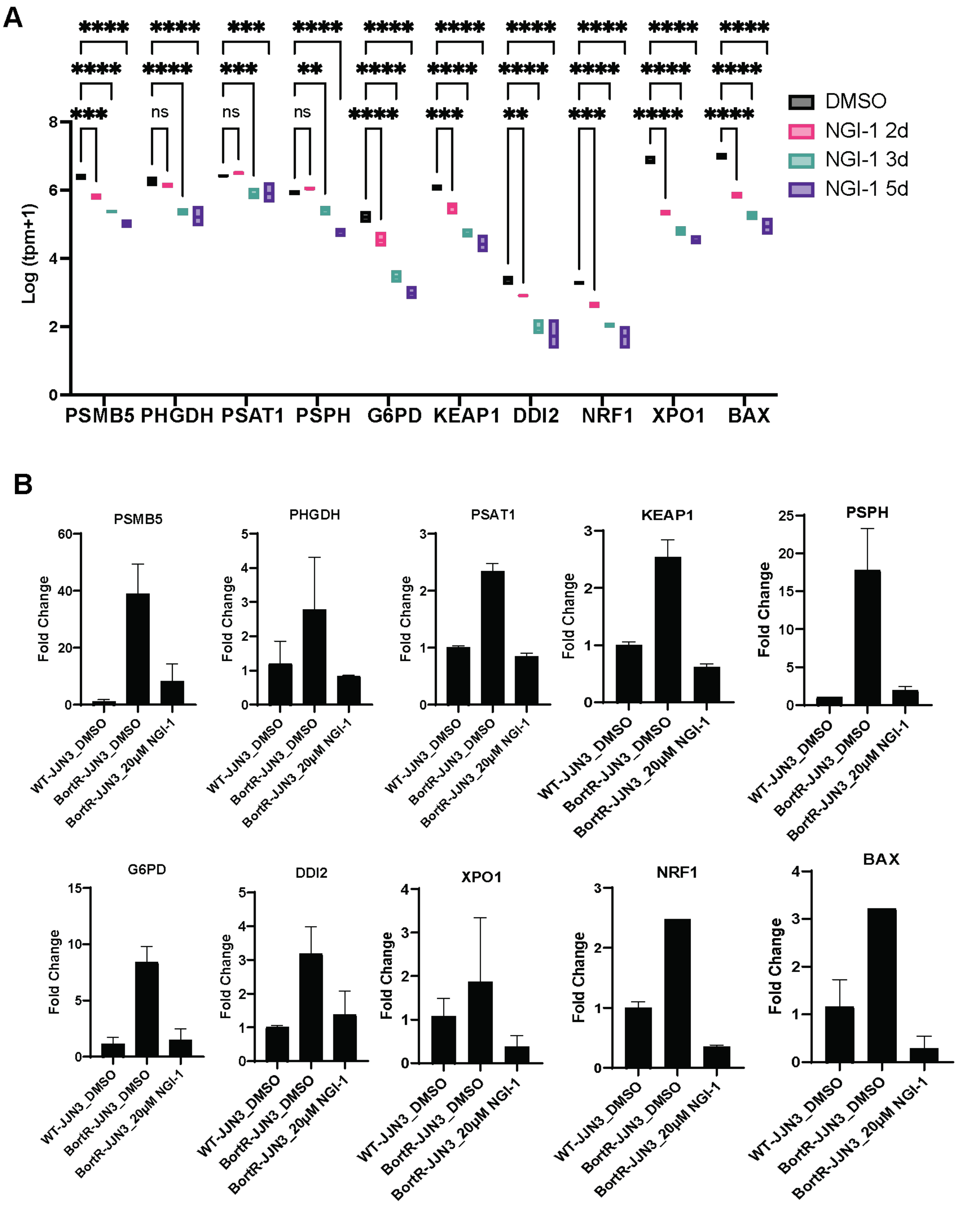

Figure S11. Expression levels of genes that are known to associated with bortezomib resistant phenotype were extracted from RNA-seq data (A) of MM1S cells treated with NGI-1 as indicated. (B) Real-time PCR data showing the expression of indicated genes in parental JJN3 and bortezomib resistant JJN3 (BortR-JJN3) cells upon treating with NGI-1.

Table S1. Antibodies used in this study		
DDOST Polyclonal antibody	14916-1-AP	Protein tech
STT3A Polyclonal antibody	12034-1-AP	Protein tech
STT3B Polyclonal antibody	15323-1-AP	Protein tech
DAD1 Polyclonal antibody	10531-1-AP	Protein tech
Beta Actin Monoclonal antibody	66009-1-Ig	Protein tech
GAPDH Monoclonal antibody	60004-1-Ig	Protein tech
HRP-conjugated Affinipure Goat Anti-Mouse IgG(H+L)	SA00001-1	Protein tech
HRP-conjugated Affinipure Goat Anti-Rabbit IgG(H+L)	SA00001-2	Protein tech
ITGB1 ANTIBODY	PA529606	Thermo
Goat Anti-Mouse IgG(H+L), Human ads-HRP	1031-05	SouthernBiotech
Goat Anti-Rabbit IgG-HRP	4030-05	SouthernBiotech
c-MYC(D84C12) Rabbit mAb	#5605	Cell signaling
S6K (49D7)	#2708	Cell Signaling
p-S6K-T389 (108D2)	#9234	Cell Signaling
p-eIF2a-S51 (D9G8)	#3398	Cell Signaling
eIF2a	#9722	Cell Signaling
CD138-APC (MI15)	#356506	Biolegend
PE/Cy7 anti-mouse CD45 (Clone 30-F11)	#103113	Biolegend
PE anti-human CD253 (TRAIL) Antibody	308206	Biolegend
